# Sounding the Alarm: Sex Differences in Rat Ultrasonic Vocalizations during Pavlovian Fear Conditioning and Extinction

**DOI:** 10.1523/ENEURO.0382-22.2022

**Published:** 2022-12-15

**Authors:** Mikaela A. Laine, Julia R. Mitchell, Johanna Rhyner, Rose Clark, Akshara Kannan, Jack Keith, MaryClare Pikus, Emmett Bergeron, Isabella Ravaglia, Ece Ulgenturk, Ashwini Shinde, Rebecca M. Shansky

**Affiliations:** Department of Psychology, College of Science, Northeastern University, Massachusetts 02115

**Keywords:** defensive behaviors, fear conditioning, SABV, ultrasonic vocalizations

## Abstract

Pavlovian fear conditioning is a prevalent tool in the study of aversive learning, which is a key component of stress-related psychiatric disorders. Adult rats can exhibit various threat-related behaviors, including freezing, motor responses, and ultrasonic vocalizations (USVs). While these responses can all signal aversion, we know little about how they relate to one another. Here we characterize USVs emitted by male and female rats during cued fear acquisition and extinction, and assess the relationship between different threat-related behaviors. We found that males consistently emitted >22 kHz calls (referred to here as “alarm calls”) than females, and that alarm call frequency in males, but not females, related to the intensity of the shock stimulus. Interestingly, 25% of males and 45% of females did not emit any alarm calls at all. Males that did make alarm calls had significantly higher levels of freezing than males who did not, while no differences in freezing were observed between female Alarm callers and Non-alarm callers. Alarm call emission was also affected by the predictability of the shock; when unpaired from a tone cue, both males and females started emitting alarm calls significantly later. During extinction learning and retrieval sessions, males were again more likely than females to emit alarm calls, which followed an extinction-like reduction in frequency. Collectively these data suggest sex dependence in how behavioral readouts relate to innate and conditioned threat responses. Importantly, we suggest that the same behaviors can signal sex-dependent features of aversion.

## Significance Statement

Behavioral neuroscientists can access various outputs during behavioral tests to draw conclusions about the internal states of animals. While freezing is the most common index of rodents feeling threatened, these animals also emit specific ultrasonic vocalizations during aversive situations. Here we record several motor and vocal behaviors to assess how they relate to each other as threat responses, and how such relationships vary across sex. We found robust differences in how much male and female rats engaged in so-called alarm vocalizations. These vocalizations were subject to extinction in both sexes but correlated with freezing only in males. As the field advances to include more females in preclinical research, it is crucial that we understand how similar-appearing outputs may reflect sex-biased features.

## Introduction

Understanding how memories of aversive situations are formed is an important goal for preclinical research on post-traumatic stress disorder and other diagnostic categories where such memories are affected (Heldt et al., 2007; de Quervain et al., 2009; Giustino and Maren, 2015). These processes have been significantly elucidated by preclinical approaches using animal models. Most notably, Pavlovian fear conditioning (FC) has long been the gold standard method for studying the acquisition and extinction of associations between aversive unconditioned stimuli (US; typically, a mild electric footshock) and previously neutral conditioned stimuli (CS; e.g., an auditory tone or a scent). After repeated CS–US paired presentations, mere exposure to the CS begins to elicit defensive behaviors (Bolles and Collier, 1976; Fanselow, 1984; Killcross et al., 1997; LeDoux, 2000). Further repeated exposure to the CS in the absence of the US typically results in extinction of the defensive behavior, a process that has been leveraged to improve exposure therapies for humans (Ressler et al., 2004; Davis et al., 2006; Hofmann et al., 2006). In fear-conditioning experiments, the typical behavioral readout of increased association between the CS and US is locomotor behavior, such as rapid darting or the complete absence of movement (i.e., freezing; Bolles and Collier, 1976; Fanselow, 1980; Blanchard et al., 1986; Gruene et al., 2015; Fadok et al., 2017; Borkar et al., 2020; Mitchell et al., 2022).

Another facet of the rodent threat response is ultrasonic vocalization (USV). USVs have been described across the rodent life span as potential indicators of emotional valence (Hofer and Shair, 1978; Knutson et al., 2002; Portfors, 2007; Wöhr and Schwarting, 2008; Takahashi et al., 2010; Reinhold et al., 2019; Granata et al., 2021; Kalenscher et al., 2021). When faced with aversive situations, such as exposure to predator odors or stressful behavioral tests (Blanchard et al., 1992; Brudzynski and Ociepa, 1992; Borta et al., 2006; Litvin et al., 2007; Fendt et al., 2018), rats emit specific low-frequency (∼22 kHz) calls, often termed “alarm calls.” As expected, fear conditioning using footshocks robustly produces alarm calls in both male and female rats from various genetic backgrounds (Wöhr et al., 2005; Schwarting et al., 2007; Dupin et al., 2019). Quantification of these calls has revealed some variations in both total amounts produced across experiments and in auditory parameters (Yee et al., 2012; Willadsen et al., 2021a). However, precise temporal patterns of call emission throughout fear conditioning and extinction, and their relationship to other threat-associated behaviors remain understudied. Such behavioral readouts represent potentially fruitful avenues for capturing a more multifaceted picture of learned fear in both sexes.

Our aim was to provide a comprehensive characterization of USVs across both conditioned fear acquisition and extinction, covering the dynamic ways in which vocalizations change across these testing sessions. Additionally, we examined how unconditioned stimulus intensity (i.e., footshock intensity) and predictability moderated behavioral readouts. The objective of this work was to expand our understanding of what USVs can tell us about affective states, and whether these patterns differ between male and female rodents. As we continue to normalize the use of female rodents in behavioral neuroscience, it is critical to know whether the same experimental measures collected for decades using only male rodents reflect the same internal states in females, or whether behavioral repertoires are sex biased (Bangasser and Cuarenta, 2021; Shansky and Murphy, 2021; Rechlin et al., 2022).

## Materials and Methods

### Animals

Male (average weight at testing: 445.87 g; total *N* = 101) and female (average weight at testing: 267.32 g; total *N* = 99) Sprague Dawley rats were purchased from a commercial breeder (Charles River) and acclimated to the vivarium for 7 d before the start of handling. The vivarium was temperature (22°C ±1°C) and humidity (40% ±10%) controlled on a 12 h light/dark cycle (lights on, 7:00 A.M.), and all rats were pair housed with *ad libitum* access to food (RMH 3000, Purina) and water (filtered tap water). Each cage contained a tinted Plexiglas chamber for nesting and enrichment, and heat-treated pine shavings for bedding. All behavioral experiments were conducted during the light phase between 9:00 A.M. and 3:00 P.M. All procedures were conducted in accordance with the National Institutes of Health *Guide for the Care and Use of Laboratory Animals* and were approved by the Northeastern University Institutional Animal Care and Use Committee.

### Behavioral data collection and analysis

**Figure 1.**
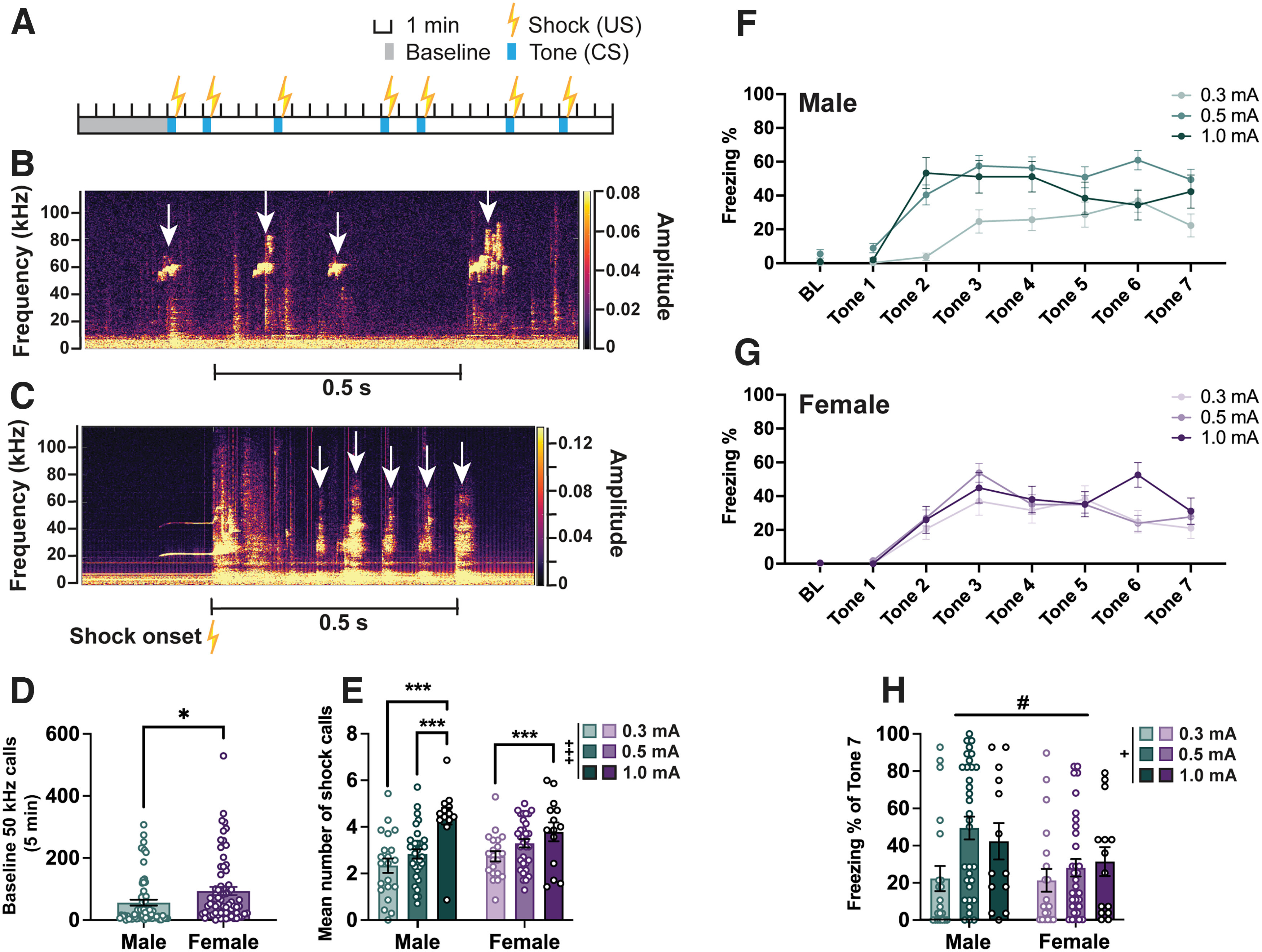
Sex differences in ultrasonic baseline and shock calls, and freezing during fear conditioning. ***A***, Schematic of cued fear conditioning showing timing and duration of tones (CS) and shocks (US). A subset of animals included here received clozapine-N-oxide (CNO) injections before fear conditioning as part of a different experiment but are analyzed together with other animals because no effect on alarm call parameters was observed (Extended Data Fig. 1-1). ***B***, Representative spectrogram (from DeepSqueak) showing typical high-frequency ultrasonic calls (white arrows) recorded during the baseline period. ***C***, Representative spectrogram showing typical shock calls (white arrows). Yellow lightning symbol denotes shock onset time. ***D***, Bar graph showing the total number of baseline 50 kHz calls emitted by male and female rats before fear conditioning. ***E***, Bar graph showing the mean number of shock calls emitted in response to each shock averaged across the trial (7 shocks) by each animal, split by sex and shock intensity. ***F***, Percentage of time male rats within each shock intensity group spent freezing during baseline (BL; first 2 min) and each tone. ***G***, Percentage of time female rats within each shock intensity group spent freezing during BL (first 2 min) and each tone. ***H***, Comparison of the freezing percentage at the end of fear conditioning (tone 7) between males and females, and across shock intensities. *N* values: ***D***: 67 males, 67 females; ***E–H***: 67 males (0.3 mA = 21, 0.5 mA = 33, 1 mA = 13), 67 females (0.3 mA = 20, 0.5 mA = 33, 1 mA = 14). Bar graphs depict the mean ± SEM, and each dot represents a single animal. Symbols along line graphs indicate the mean ± SEM. Significant main effects of shock intensity (+) and sex (#), and *post hoc*/pairwise comparisons (*) are denoted with different symbols, with 1 (*p *<* *0.05), 2 (*p *<* *0.01), or 3 (*p *<* *0.001) symbols depicting the degree of significance.

**Figure 2.**
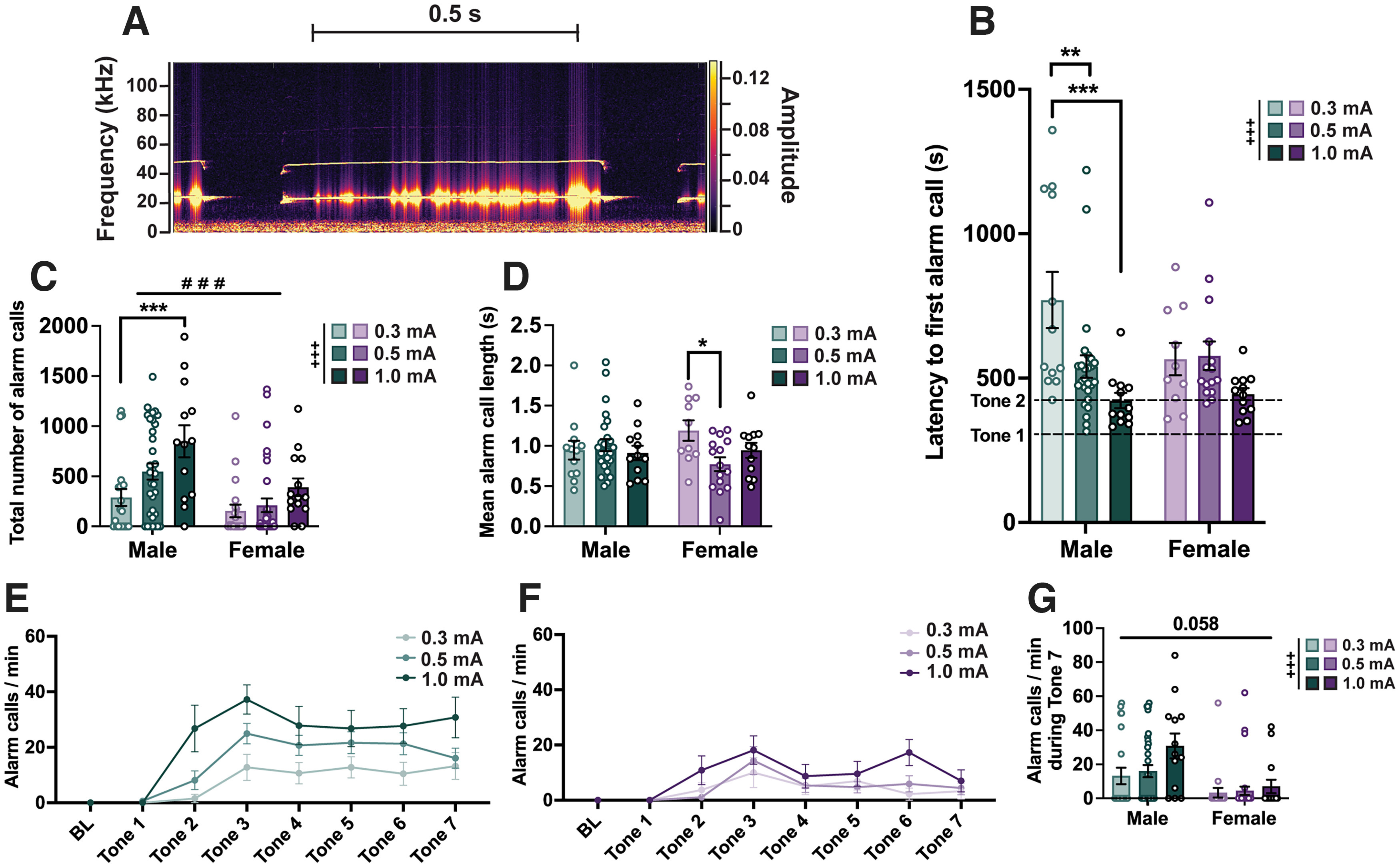
Sex differences in ultrasonic alarm calls during fear conditioning. ***A***, Representative spectrogram (from DeepSqueak) showing an ultrasonic alarm call captured during an intertrial interval, with a principal frequency of ∼22 kHz. Alarm calls were not observed in animals exposed only to the experimental context and tones (Extended Data Fig. 2-1). ***B***, Bar graph depicting the latency of each animal to emit their first alarm call, split across sex and shock intensity. Dashed lines indicate the timing of the first and second tone starts. ***C***, Bar graph depicting the total number of alarm calls emitted during a fear-conditioning trial, split across sex and shock intensity. ***D***, Bar graph depicting the mean alarm call length, split across sex and shock intensity. ***E***, Line graph showing the normalized (per minute) alarm call rate of male rats across shock intensity groups during baseline (BL; 5 min) and each tone. ***F***, Line graph showing the normalized (per minute) alarm call rate of female rats across shock intensity groups during BL (5 min) and each tone. ***G***, Comparison of alarm call rate at the end of fear conditioning (tone 7) between males and females, and across shock intensities. *N* values: ***C***, ***E–G***: 67 males (0.3 mA = 21, 0.5 mA = 33, 1 mA = 13), 67 females (0.3 mA = 20, 0.5 mA = 33, 1 mA = 14); ***B***, ***D***: 50 males (0.3 mA = 12, 0.5 mA = 26, 1 mA = 12), 37 females (0.3 mA = 10, 0.5 mA = 15, 1 mA = 12; Non-alarm callers excluded; Fig. 5, analysis of Alarm callers vs Non-alarm callers). Bar graphs depict the mean ± SEM, and each dot represents a single animal. Symbols along line graphs indicate the mean ± SEM. Significant main effects of shock intensity (+) and sex (#), and *post hoc* comparisons (*) are denoted with different symbols, with 1 (*p *<* *0.05), 2 (*p *<* *0.01), or 3 (*p *<* *0.001) symbols depicting the degree of significance.

Rats were handled on 2 d before cued fear conditioning and habituated to transport from the vivarium to the testing room on a trolley the day before conditioning. On the conditioning day, rats were brought in 30 min before the start to habituate to the testing room (<15 m from the vivarium) and ambient noise. They were then placed in sound-attenuating conditioning chambers (model H10-24A Rat Test Cage, Coulbourn Instruments), consisting of Plexiglas, metal walls, and a metal grid floor for the delivery of footshocks (model H10-11R-TC Shock Floor, Coulbourn Instruments). The chambers were dimly lit by an overhead light (2 lux). Following a 5 min baseline period with no stimulus presentations, the rats were sequentially exposed to a total of seven CS–US pairings (Fig. 1*A*). As CS, we used a 30 s 4 kHz tone played in each chamber by a speaker at ∼75 dB as measured at the center of the chamber (∼20 cm from the speaker). For paired fear conditioning [Figs. 1, 2 (also see Figs. 4-7)] the footshock US, lasting 0.5 s, was presented at the end of the tone, with the two stimuli coterminating. Each rat received footshocks representing one of the following three shock intensities: 0.3 mA (mild shock intensity), 0.5 mA (moderate shock intensity), or 1 mA (high shock intensity). The intertrial interval (ITI) was of varying lengths between each of the CS–US pairings (90–330 s). Two to four rats were conditioned simultaneously in the same room, without mixing sexes within each run. Chambers and the testing cages were cleaned with water and ethanol between each run. See figure legends for exact *N* values for each group.

10.1523/ENEURO.0382-22.2022.f1-1 Figure 1-1 CNO exposure does not affect alarm call parameters in males or females. ***A****–****E***, Bar graphs depicting the total number of alarm calls emitted during fear conditioning (***A***), the latency to the first alarm call during the session (***B***), the number of all calls emitted during the baseline period (***C***), the mean alarm call length (***D***), and the average number of shock calls emitted per animal across all 7 shocks (***E***) of male and female rats exposed to 0.5 mA footshocks, compared between experiments (one involving CNO injection and one with no injections). ***F***, Bar graphs depicting the average maximum velocity reached immediately after shock delivery, split by sex and shock intensity. Bar graphs depict the mean ± SEM, and each dot represents a single animal. Significant main effects of shock intensity (+) and sex (#), and *post hoc* comparisons (*) are denoted with different symbols, with 1 (*p* < 0.05), 2 (*p* < 0.01), or 3 (*p* < 0.001) symbols depicting the degree of significance. Download Figure 1-1, file.

10.1523/ENEURO.0382-22.2022.f2-1 Figure 2-1 Mere exposure to the experimental context and tone are not sufficient to produce alarm calls, and shock-driven alarm calls are also observed during the ITIs. ***A–C***, Bar graph showing the total number of USVs across the whole trial (***A***), the total number of USVs emitted during the baseline (***B***), and the total number of alarm calls (***C***) emitted by male and female rats exposed to the same testing procedure, chamber, and tones as those in Figure 1, but with no footshocks. ***D***, Line graph showing the percentage of time spent freezing during baseline (first 2 min only) and each tone of animals exposed to only the tones without shocks. ***E***, Line graph depicting the rate of alarm calling of male and female rats in each shock intensity group (0.3, 0.5, and 1 mA) during the ITIs. ***F***, Line graph showing the mean alarm call length as measured during each tone and ITI, separately for males and females. *N* values: ***A–D***: 13 males, 12 females; ***E***: 67 males (0.3 mA, 21; 0.5 mA, 33; 1 mA, 13), 67 females (0.3 mA, 20; 0.5 mA, 33; 1 mA, 14); ***F***: 50 males, 37 females (non-alarm callers excluded). Bar graphs depict the mean ± SEM, and each dot represents a single animal. Symbols along line graphs indicate the mean ± SEM. Download Figure 2-1, file.

**Figure 3.**
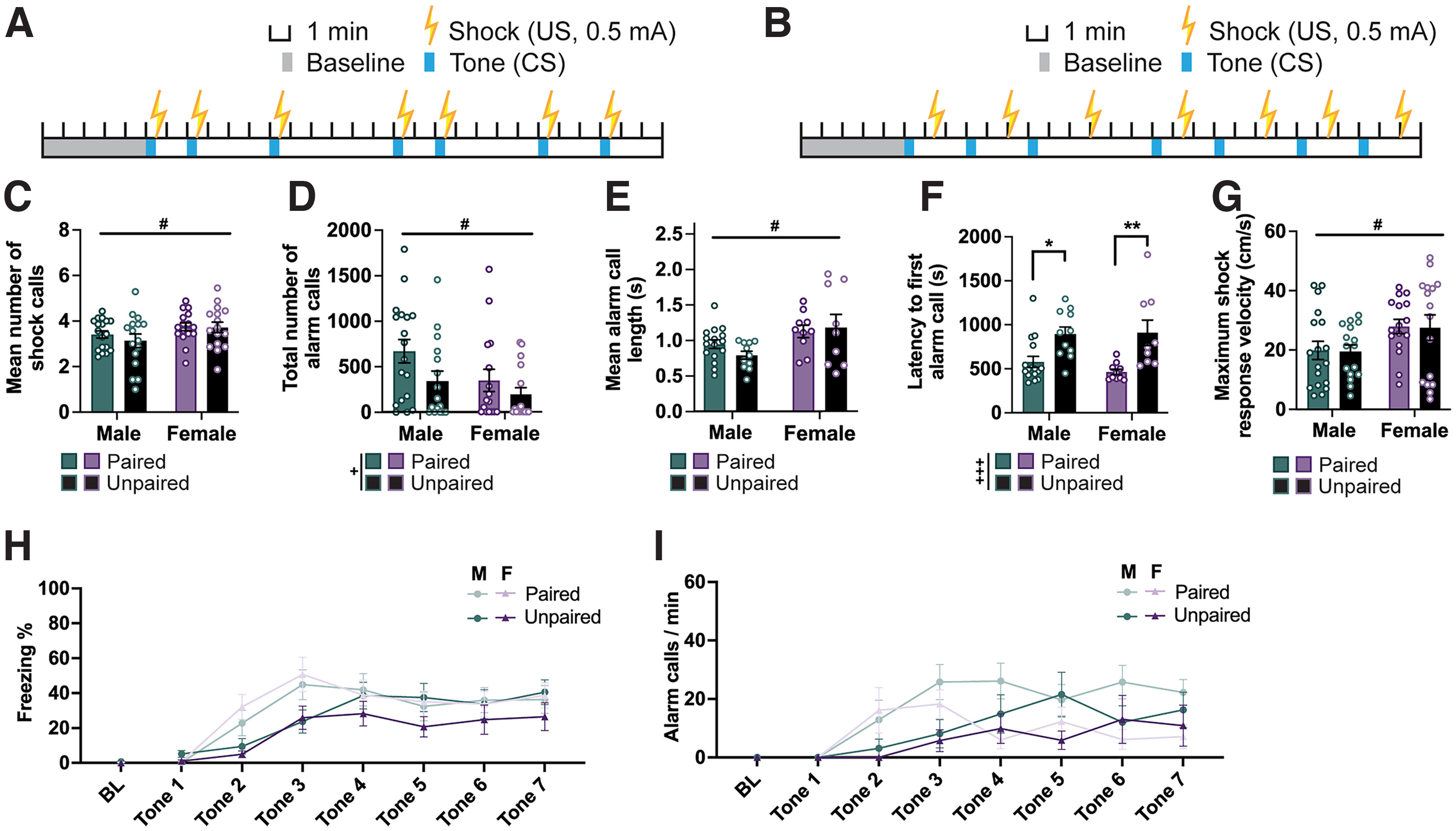
Unpaired CS and US results in delayed latency to alarm call. ***A***, ***B***, Schematics of cued fear conditioning showing timing and duration of tones (CS) and shocks (US) for paired (***A***) and unpaired (***B***) protocols. All animals in this cohort received 0.5 mA footshocks. ***C***, Bar graph showing the mean number of shock calls emitted in response to each shock averaged across the trial (7 shocks) by each animal. ***D***, Bar graph depicting the total number of alarm calls emitted during a fear-conditioning trial. ***E***, Bar graph depicting the mean alarm call length. ***F***, Bar graph depicting the latency of each animal to emit their first alarm call. ***G***, Bar graph depicting the maximum velocity reached in response to each shock averaged across all 7 shocks within a trial by each animal. ***H***, Line graph showing the percentage of time male and female rats within each protocol group (paired vs unpaired) spent freezing during baseline (BL; first 2 min) and each tone. ***I***, Line graph showing the normalized (per minute) alarm call rate of male and female rats within each protocol group (paired vs unpaired) during BL (5 min) and each tone. *N* values: ***C***, ***D***, ***G–I***: paired: 18 males, 16 females; unpaired: 16 males, 16 females; ***E***, ***F***: Paired: 16 males, 10 females; Unpaired, 11 males, 9 females (Non-alarm callers excluded). Bar graphs depict the mean ± SEM, and each dot represents a single animal. Symbols along line graphs indicate the mean ± SEM. Significant main effects of pairing (+) and sex (#), and *post hoc* comparisons (*) are denoted with different symbols, with 1 (*p *<* *0.05), 2 (*p *<* *0.01), or 3 (*p *<* *0.001) symbols depicting the degree of significance.

A distinct cohort of animals was part of an experiment to compare exposure to unpaired (16 males,  16 females) or paired (18 males, 16 females) fear conditioning (Fig. 3). The parameters of the CS and US were identical to paired fear conditioning, aside from the shock always occurring a minimum of 60 s after the cessation of the tone. The ITIs of CS and US presentations were varied (Fig. 3*A*).

Stimulus delivery was controlled, and videos of the animals’ behavior were recorded using Ethovision (version 16; Noldus Technologies) and infrared digital cameras mounted on top of each conditioning chamber. Time spent freezing, the occurrence of darts, and the maximum velocity the rat reached as a response to the shock were analyzed using freely available Python-based software ScaredyRat (Mitchell et al., 2022). An animal was classified as a Darter if it performed one or more darts (movement at a speed exceeding 20 cm/s) during one or more tones (excluding the first two tones as well as the immediate response to the shock). For all fear-conditioning trials, baseline freezing was recorded from the first 2 min of the 5 min stimulus-free time in the chamber. Freezing was defined in ScaredyRat as the absence of observable movement, with a minimum bout duration of 1 s.

Our dataset includes animals (20 males, 20 females) that were part of a different project involving systemic injections of clozapine-*N*-oxide (CNO) for circuit-specific activation using designer receptors activated by designer drugs. These rats underwent intracranial infusions of viral vectors (AAV_retro_ pmSyn1-EBFP-Cre, and pAAV_8_-hSyn-DIO-hM4D(Gi)-mCherry, pAAV_8_-hSyn-DIO-hM3D(Gq)-mCherry, or pAAV_8_-hSyn-DIO-mCherry; all sourced from Addgene) under isoflurane anesthesia, allowing 5–6 weeks of recovery before behavioral testing. However, most (35 of 40) of these animals did not show any fluorescent signal marking viral expression. To ensure that this experience did not influence behavioral outcomes, we compared these animals to same-sex animals receiving the same footshock intensity (0.5 mA), but no surgical experience or CNO exposure. This analysis revealed no differences in alarm call rate, length, or latency, or in shock call rate (Extended Data Fig. 2-1), thus justifying their inclusion in the dataset. These animals were also exposed to extinction learning (EL) and extinction retrieval (EL; see Fig. 6). Extinction learning was conducted 24 h after fear conditioning, and extinction retrieval 24 h after extinction learning. For both tests, animals were brought into the testing room 30 min before the start of the test to habituate. The testing room and chambers were the same as for fear conditioning, but with different lighting (8 lux), scent (2–4 drops of Dr Bronner’s peppermint-scented pure-castile liquid soap placed on a train underneath the test cage floor) and chamber features (black Plexiglas floor covering the metal grids). For extinction learning, baseline behavior was recorded for the full 5 min of stimulus-free time before the start of tone presentations. For these experiments, we included the full baseline duration because we observed that animals that emitted alarm calls before tone start frequently did so also at the end of the baseline period. Thus, to be able to compare freezing and alarm calling, we chose to record both for the full duration. Animals received a total of 20 presentations of the same tone CS they heard during fear conditioning, at varying intervals (90–330 s), with no shocks. Similarly, for extinction retrieval 24 h after extinction learning the animals were exposed to a baseline period of 5 min followed by three presentations of the same tone at varying intervals (150–240 s). Time spent freezing in extinction learning and retrieval was quantified using a combination of ScaredyRat and hand scoring by trained investigators (each tone evaluated by two independent investigators and their scores averaged; where the scores differed by >2 s, a third investigator resolved the discrepancy), because of animals often falling asleep, particularly during the latter tones of extinction learning. Motion-tracking methods such as that used here do not distinguish sleeping and freezing, and thus hand scoring of these trials is warranted.

### USV recording and analysis

Throughout the behavioral experiments, we recorded vocalizations emitted in the audible and ultrasonic range (0–120 kHz; sampling rate, 250 kHz) using microphones (model CM16, Avisoft Bioacoustics) mounted over each conditioning chamber. Pilot testing showed that each microphone was able to detect sounds only from the chamber it was in; no cross-detection from other chambers was observed (data not shown). The audio files were processed with DeepSqueak (version 3; Coffey et al., 2019), a publicly available user interface that uses machine learning to detect spectrograms typical of rodent USVs. All detected calls were manually confirmed by a trained investigator. Temporal alignment of the audio and behavioral data was confirmed by observation of the tone cue within the audible range of the spectrogram. We then aligned each detected call with an epoch (baseline, tone, shock, or ITI) and identified alarm calls based on specific criteria (call length, ≥70 ms; main frequency of the call, ≤30 kHz; change in frequency, ≤10 kHz).

Group differences were analyzed using SPSS (version 27) and GraphPad Prism (version 8). The specific test used was determined by data type and structure (see Results for details). Geisser–Greenhouse correction for nonsphericity was applied when needed. Correlation analyses (see Figs. 6, 7) were conducted in SPSS using Pearson’s method if both variables in the analysis were normally distributed (determined using the Shapiro-Wilk test), and Spearman’s method if one or both were non-normally distributed. Bonferroni correction was used to adjust *p*-values for multiple comparisons where appropriate, and an α level of 0.05 was used throughout. Outlier data points were excluded based on the ROUT method in GraphPad Prism (FDR, *q *=* *0.1).

## Results

### Sex differences in short ultrasonic and audible calls during fear conditioning as a function of shock intensity

To investigate the nature of USVs occurring during cued fear conditioning (Fig. 1*A*), we recorded and analyzed auditory data ranging from 0 to 120 kHz. During the baseline period, while the rats explore the conditioning apparatus before any tones or shocks, animals frequently engage in various types of chatter in the form of short high-frequency (50 kHz) calls (Fig. 1*B*). Females emitted more such calls than males (independent *t* test: *t *=* *2.355, *p *=* *0.0200; Fig. 1*D*). Another specific type of call spanning audible and ultrasonic frequencies was observed when the animals were given the footshocks, and these will be referred to as “shock calls” (Fig. 1*C*). To assess how well the shock calls reflect the potential degree of discomfort the animals experience, we asked whether shock intensity influences their occurrence. A two-way ANOVA suggested a significant main effect of shock intensity (*F*_(2,128)_ = 14.36, *p *<* *0.001), with both males and females receiving 1 mA footshocks emitting the highest number of these calls (males: 0.3 vs 1 mA; *p *<* *0.001; males: 0.5 vs 1 mA; *p *<* *0.001; females: 0.3 vs 1 mA; *p *=* *0.040; Fig. 1*E*).

In line with past research, we observe robust freezing behavior across the fear-conditioning trial in both males and females (Fig. 1*F*,*G*). At the end of the trial (during tone 7) females froze significantly less than males (two-way ANOVA; main effect of sex: *F*_(1,128)_ = 3.995, *p *=* *0.048; Fig. 1*H*). Additionally, the main effect of shock intensity on freezing during tone 7 was significant (*F*_(2,128)_ = 4.034, *p *=* *0.020), with a significant *post hoc* contrast only in male 0.3 versus 0.5 mA group comparison (*p *=* *0.006).

### Sex differences in alarm calls during fear conditioning as a function of shock intensity

A distinct type of USV is emitted when rats experience aversive events, such as exposure to fear conditioning (Burgdorf et al., 2000; Wöhr et al., 2005; Borta et al., 2006; Yee et al., 2012) or a context associated with a predator odor (Fendt et al., 2018) in adults. These calls are relatively long, with a principal frequency of 22 kHz (Fig. 2*A*), and will be referred to here as alarm calls. Animals exposed only to handling, the experimental apparatus, and tones without footshocks do not emit alarm calls, suggesting novelty and the stress of performing the experiment alone are not sufficient to elicit them (Extended Data Fig. 2-1). Typically, these calls were not emitted until after the second tone–shock pairing, although males receiving 0.5 or 1 mA footshocks started emitting alarm calls earlier than those receiving 0.3 mA shocks (two-way ANOVA; main effect of shock intensity: *F*_(2,81)_ = 8.092, *p *<* *0.001; *post hoc* comparisons: male: 0.3 vs 0.5 mA; *p *=* *0.004; male: 0.3 vs 1 mA; *p *<* *0.001; Fig. 2*B*). Overall, males made significantly more alarm calls than females (two-way ANOVA; main effect of sex: *F*_(1,128)_= 16.67; *p *<* *0.001; main effect of shock intensity: *F*_(2,128)_ = 7.622; *p *<* *0.001), and males receiving strong footshocks made more alarm calls than those receiving mild shocks (Fig. 2*C*). Alarm calls occurred consistently also during the ITIs, and they were of similar length between epochs across the whole trial (Extended Data Fig. 2-1). Mean alarm call length did not differ between sexes, but there was a significant interaction between sex and shock intensity (two-way ANOVA; *F*_(2,81)_ = 3.226; *p *=* *0.045) driven by females in the 0.5 mA condition making significantly shorter alarm calls than those in the 0.3 mA condition (*p *=* *0.018; Fig. 2*D*). The pattern of alarm calling across the fear-conditioning session mirrors that of freezing (Fig. 1*F–H*), and at the last tone there was a significant main effect of shock intensity with stronger shocks associating with higher alarm call rates (two-way ANOVA; *F*_(1,128)_ = 19.53; *p *<* *0.001) and a trend toward a main effect by sex (males emitting more alarm calls than females: *F*_(2,128)_ = 2.912; *p *=* *0.058; Fig. 2*E–G*).

### Unpredictability of the shock drives delay in initiation of alarm calls

Next, we asked whether predictability of the US affects the nature of USVs by comparing male and female rats exposed to paired (coterminating US and CS; Fig. 3*A*) and unpaired (independently occurring US and CS; Fig. 3*B*) fear conditioning (shock intensity, 0.5 mA for all). Rats exposed to unpaired fear conditioning did not differ from those exposed to paired fear conditioning on measures of the number of shock calls (Fig. 3*C*), alarm call length (Fig. 3*E*), or maximum shock response velocity (Fig. 3*G*), as assessed by two-way ANOVA. However, on each of these measures we observed a main effect of sex, with female rats making more shock calls (*F*_(1,62)_ = 4.092; *p *=* *0.047), and longer alarm calls (*F*_(1,62)_ = 5.235; *p *=* *0.027), and moving faster after the shock (*F*_(1,62)_ = 6.825; *p *=* *0.011). We found a main effect of pairing condition on both the total number of alarm calls emitted and the latency to alarm; rats in the unpaired condition emitted overall fewer alarm calls (main effect of pairing: *F*_(1,62)_ = 4.608; *p *= 0.036; main effect of sex: *F*_(1,62)_ = 4.625; *p *=* *0.035; Fig. 3*D*), possibly because of their longer first-alarm call latencies (*F*_(1,62)_ = 20.500; *p *<* *0.001; Fig. 3*F*,*I*). The effect on latency is unlikely to be explained by the variations in the timing of shock delivery between pairing conditions (Fig. 3*A*,*B*). Both males and females in the paired condition on average start emitting alarm calls after the second shock occurring at 7.5 min (average latency: males, 9.6 min; females, 7.6 min), while in the unpaired condition both start emitting alarm calls after the third shock occurring at 14 min (average latency for both sexes, 14.9 min). In other words, on average, one additional shock exposure was required to elicit alarm calls in the unpaired versus paired condition. Interestingly, there is also a delay in reaching peak freezing in the unpaired condition compared with the paired one (Fig. 3*H*).

### Darting does not associate with differences in USVs

**Figure 4.**
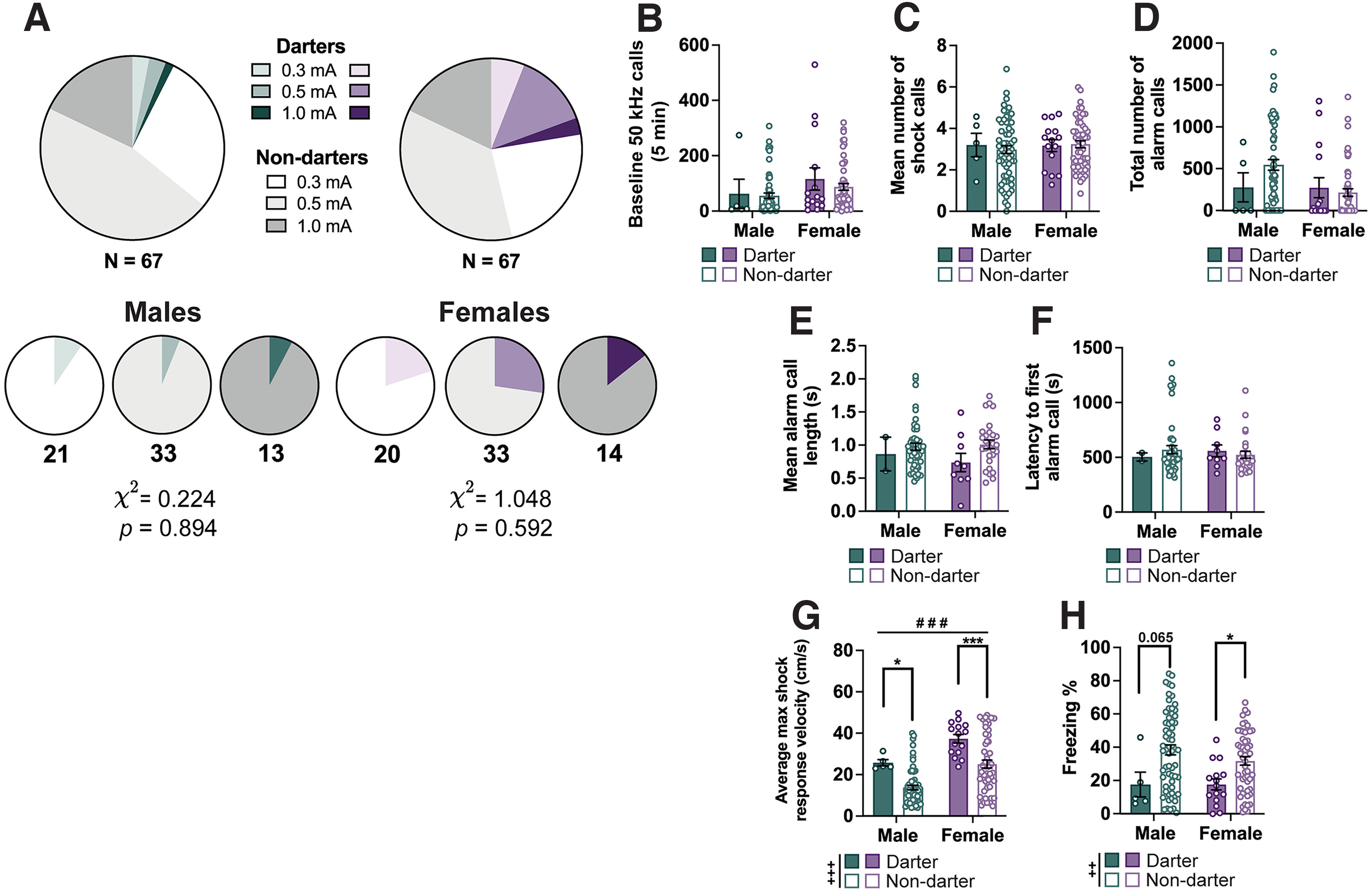
Darting does not associate with differences in USV features in either sex. ***A***, Pie charts showing the proportion of Darters and Non-darters across the whole male and female cohorts (top row) and separately for each shock intensity group (bottom row). Numbers underneath each chart denote the number of animals included within the chart, and the χ^2^ statistics for the effect of shock intensity on Darter group separately for males and females. ***B***, Bar graph showing the total number of baseline 50 kHz calls emitted by male and female Darters and Non-darters before fear conditioning. ***C***, Bar graph showing the mean number of shock calls emitted in response to each shock averaged across the trial (7 shocks) by each animal. ***D***, Bar graph depicting the total number of alarm calls emitted during a fear-conditioning trial. Two-way ANOVA suggests no significant main effects or interactions. ***E***, Bar graph depicting the mean alarm call length. ***F***, Bar graph depicting the latency of each animal to emit their first alarm call. ***G***, Bar graph depicting the maximum velocity reached in response to each shock averaged across all 7 shocks within a trial by each animal. ***H***, Bar graphs showing the percentage of time animals spent freezing across all 7 tones of a trial. *N* values: ***B–H***: Males: 5 Darters, 62 Non-darters; Females: 15 Darters, 52 Non-darters. Bar graphs depict the mean ± SEM, and each dot represents a single animal. Significant main effects of darting (+) and sex (#), and *post hoc* comparisons (*) are denoted with different symbols, with 1 (*p *<* *0.05), 2 (*p *<* *0.01), or 3 (*p *<* *0.001) symbols depicting degree of significance.

While freezing is to date the most commonly quantified index of learning the CS–US association, it is by no means the only available and informative motor behavior rodents engage in. Darting refers to rapid movements occurring during the CS, particularly after a number of CS–US pairings have been established (Gruene et al., 2015; Greiner et al., 2019; Hersman et al., 2020; Mitchell et al., 2022). We classified all animals as either Darters or Non-darters based on the occurrence of one or more darts (movement exceeding a speed of 20 cm/s) during tones 3–7 (Gruene et al., 2015; Colom-Lapetina et al., 2019; Mitchell et al., 2022), and observed Darters across all shock intensities (males: χ^2^ = 0.224; *p *=* *0.894; females: χ^2^ = 1.048; *p *=* *0.592; Fig. 4*A*). We observed more Darters among females than males (χ^2^ = 5.877; *p *=* *0.015). Two-way ANOVAs were conducted to investigate whether Darters differed from Non-darters within sex on USV variables (baseline 50 kHz calls, shock calls, alarm calls, alarm call length, and alarm call latency; Fig. 4*B–F*), and no significant main effects or interactions were observed (*p* values* *>* *0.1701). By contrast, other motor behaviors (maximum velocity reached in response to the shock, freezing) differed significantly as a function of darting. Both male and female Darters had faster maximum shock response velocities (main effect of darting: *F*_(1,130)_ = 15.93, *p *<* *0.001; male Darters vs Non-darters *p *=* *0.043; female Darters vs Non-darters *p *<* *0.001), in addition to a main effect of sex (*F*_(1,130)_ = 14.21; *p *<* *0.001; Fig. 4*G*). There was a significant main effect of darting on freezing across the fear-conditioning trial (*F*_(1,130)_ = 9.428; *p *=* *0.003), with female Darters freezing significantly less than female Non-darters (*p *=* *0.043) and a trend in the same direction for males (*p *=* *0.065; Fig. 4*H*).

### Alarm calling distinguishes high and low freezing only in males

**Figure 5.**
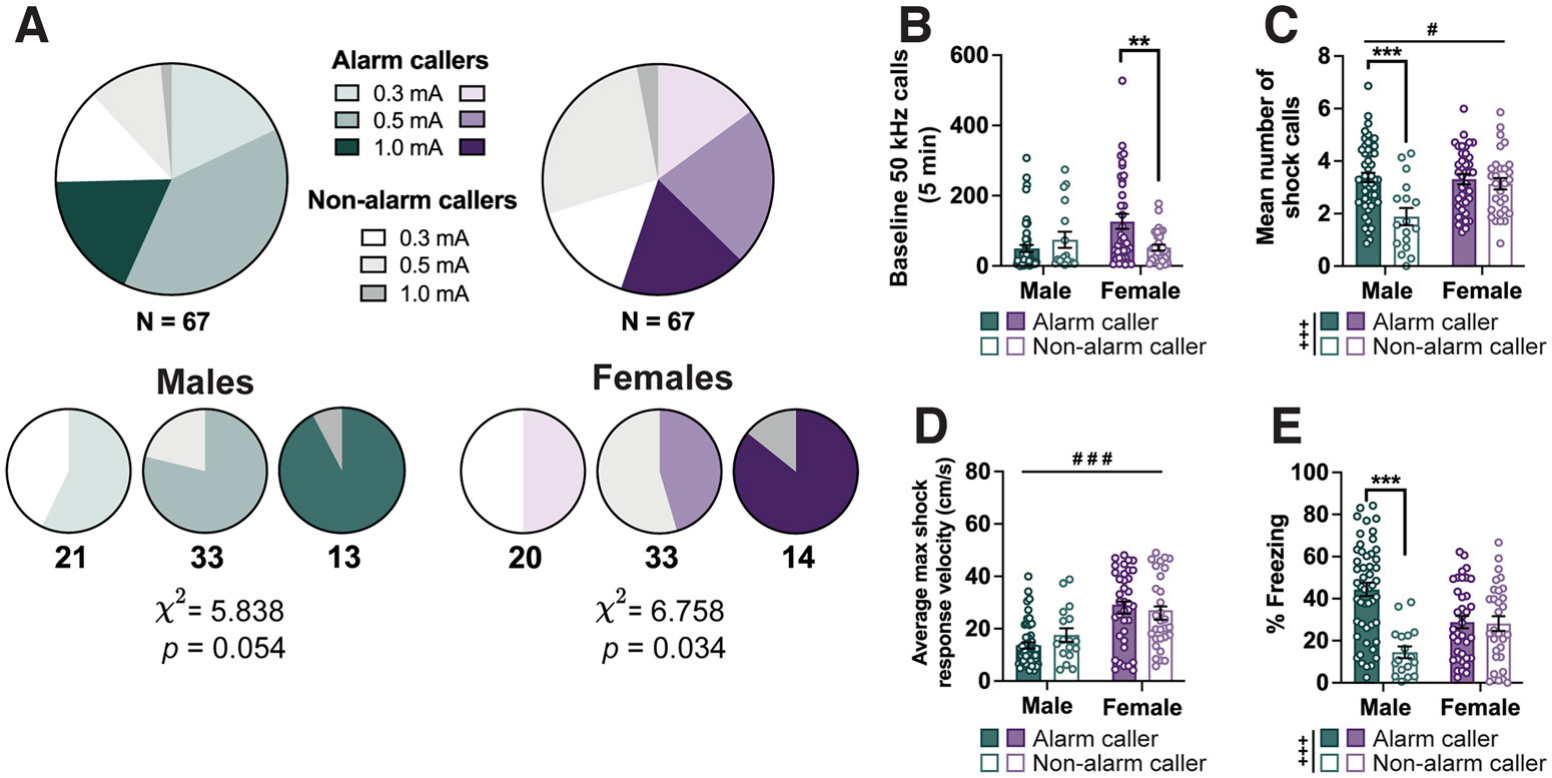
Tendency to emit alarm calls as a dichotomous phenotype associates with freezing in males only. ***A***, Pie charts showing the proportion of Alarm callers and Non-alarm callers across the whole male and female cohorts (top row) and separately for each shock intensity group (bottom row). Numbers underneath each chart denote the number of animals included within the chart and the χ^2^ statistics for the effect of shock intensity on alarm call group separately for males and females. ***B***, Bar graph showing the total number of baseline 50 kHz calls emitted by male and female Alarm callers and Non-alarm callers before fear conditioning. ***C***, Bar graph showing the mean number of shock calls emitted in response to each shock averaged across the trial (7 shocks) by each animal. ***D***, Bar graph depicting the maximum velocity reached in response to each shock averaged across all 7 shocks within a trial by each animal. ***E***, Bar graphs showing the percentage of time animals spent freezing across all 7 tones of a trial. Removing Non-alarm callers did not significantly alter the findings presented in Figure 2 (Extended Data Fig. 5-1). *N* values: ***B–E***: Males: 50 Alarm callers, 17 Non-alarm callers; Females: 37 Alarm callers, 30 Non-alarm callers. Bar graphs depict the mean ± SEM, and each dot represents a single animal. Significant main effects of alarm caller status (+) and sex (#), and *post hoc* comparisons (*) are denoted with different symbols, with 1 (*p *<* *0.05), 2 (*p *<* *0.01), or 3 (*p *<* *0.001) symbols depicting the degree of significance.

We noted that in addition to interindividual variability in alarm call rate, some rats did not emit any alarm calls during the whole fear-conditioning trial. To explore whether this behavior constituted a behavioral phenotype, we compared alarm call-emitting rats (Alarm callers) to rats that did not emit a single alarm call (Non-alarm callers) on other USV and motor behaviors. A χ^2^ test indicated that shock intensity groups differed in the frequency of Alarm callers (Fig. 5*A*), with a significant effect in females (χ^2^ = 6.758; *p *=* *0.034) and a trend in males (χ^2^ = 5.838; *p *= 0.054). In both sexes, we observed more Alarm callers in high-shock intensity groups (1 mA) than the lower-shock intensity groups. We also replicated the alarm call analyses depicted in Figure 2 after excluding rats that did not emit any alarm calls, and the key findings remained unchanged (Extended Data Fig. 5-1). While the data shown in Figure 4 are collapsed across shock intensity and analyzed by two-way ANOVA (sex × alarm calling), we also performed linear regression analysis to evaluate the contribution of shock intensity to group differences (sex, shock intensity, and alarm calling as predictors). First, we found a significant sex × alarm calling interaction effect on the number of baseline calls (*F*_(1,130)_ = 8.933; *p *=* *0.003; Fig. 5*B*), with a significant pairwise comparison only in females (*p *=* *0.002) suggesting that Alarm callers are more vocal during the baseline period. In the linear regression model, sex was the strongest and the only significant predictor of baseline calling (β = 0.230; *p *=* *0.009), with a trend toward a significant contribution of alarm calling (β = −0.149; *p *=* *0.100). Next, a two-way ANOVA suggests main effects of both sex and alarm calling on the number of shock calls (main effect of sex: *F*_(1,130)_ = 6.517; *p *=* *0.012; main effect of alarm calling: *F*_(1,130)_ = 12.820; *p *<* *0.001, interaction: *F*_(1,130)_ = 7.891; *p *=* *0.006; Fig. 5*C*), with a significant pairwise comparison only in males where Alarm callers emitted significantly more shock calls (*p *<* *0.001). The linear regression analysis suggests that a larger portion of this effect was attributable to shock intensity (β = 0.371; *p *<* *0.001) than alarm calling (β = −0.166; *p *=* *0.047), although both served as significant predictors. No significant differences were observed between Alarm callers and Non-alarm callers on maximum velocity in response to the shock (Fig. 5*D*). Interestingly, when analyzing the average percentage of the CS duration the rats spent freezing, we found a significant main effect of alarm calling (*F*_(1,130)_ = 17.91; *p *<* *0.001; sex × alarm calling interaction: *F*_(1,130)_ = 16.32; *p *<* *0.001) and a significant pairwise comparison within males (*p *<* *0.001), suggesting that rats that did not make any alarm calls also froze considerably less than their conspecifics that did emit alarm calls. Linear regression suggests that this effect could not be attributed to shock intensity (β = 0.106; *p *=* *0.213), but was significantly affected by alarm calling (β = −0.266; *p *=* *0.003).

10.1523/ENEURO.0382-22.2022.f5-1 Figure 5-1 Removing non-alarm callers does not eliminate the sex difference in alarm call rate. ***A***, Total number of alarm calls emitted by rats included in Figure 2*C*, excluding rats that did not make any alarm calls. ***B***, ***C***, Line graphs showing the normalized (per minute) alarm call rate of male (***B***) and female (***C***) rats across shock intensity groups during baseline (BL; 5 min) and each tone, excluding rats that did not make any alarm calls. *N* values: 50 males (0.3 mA, 12; 0.5 mA, 26; 1 mA, 12), 37 females (0.3 mA, 10; 0.5 mA, 15; 1 mA, 12). Bar graphs depict the mean ± SEM, and each dot represents a single animal. Symbols along line graphs indicate the mean ± SEM. Significant main effects of shock intensity (+) and sex (#), and *post hoc* comparisons (*) are denoted with different symbols, with 1 (*p *<* *0.05), 2 (*p *<* *0.01), or 3 (*p *<* *0.001) symbols depicting the degree of significance. Download Figure 5-1, TIF file.

### Alarm calls are extinguished across sexes, but correlate with freezing more strongly in males

**Figure 6.**
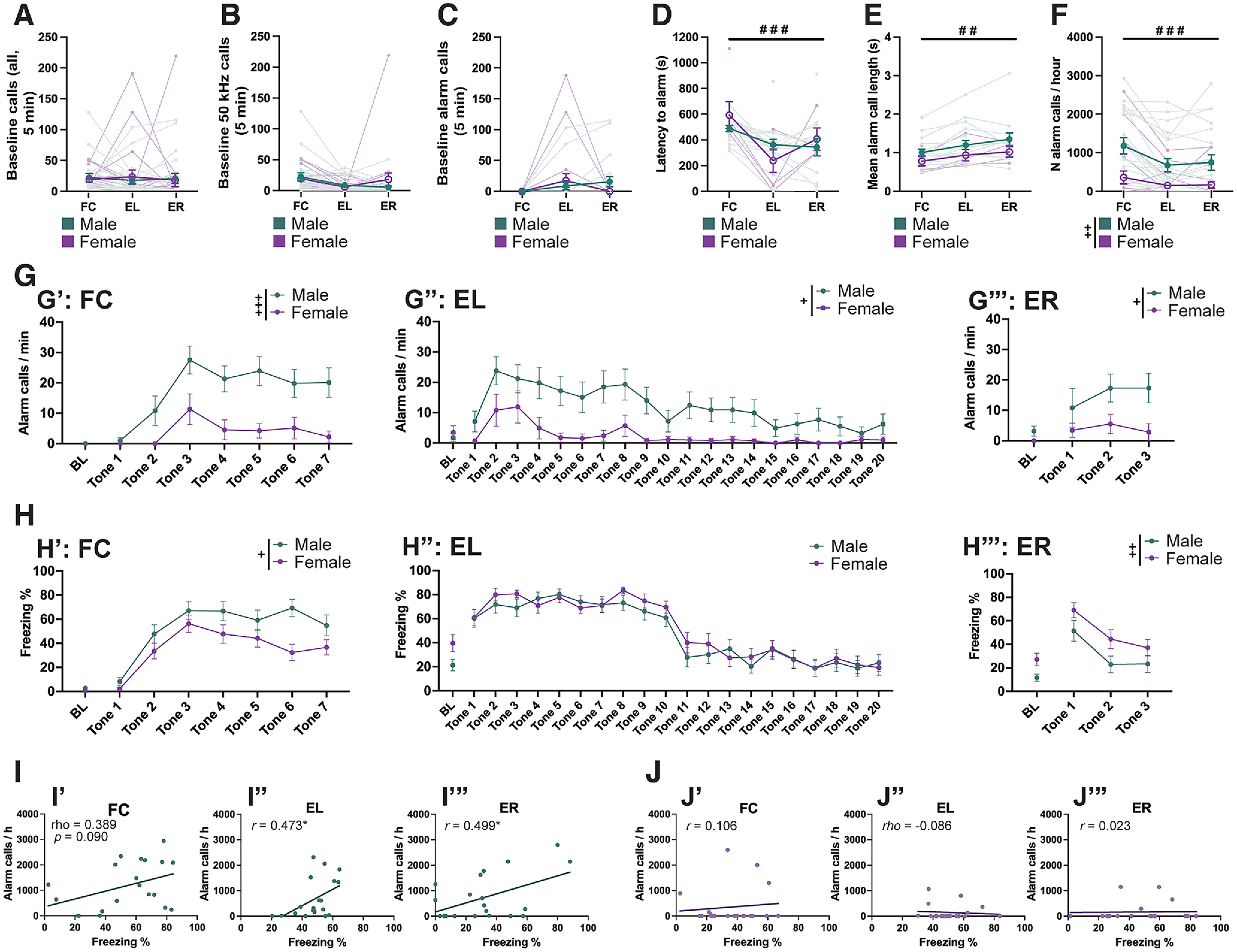
Sex differences in extinction of alarm calling. ***A–F***, Connected scatter plots of the number of all USVs (***A***), all 50 kHz calls (***B***) and all alarm calls (***C***) emitted during baseline, the latency to first alarm (***D***), the mean alarm call length (***E***), and the rate of alarm calls normalized to 1 h (***F***) of male and female rats during fear conditioning (FC), extinction learning (EL), and extinction retrieval (ER). Light colors represent individual animals, while dark colors with error bars represent sex means. ***G***, The rate of alarm calls, normalized per 1 min, during the baseline (BL) and each tone presentation during FC (***G*′**), EL (***G*′′**), and ER (***G*′′′**), shown separately for males and females. ***H***, Percentage of time spent freezing during the first 2 min of BL for FC (***H*′**), and the first 5 min of BL for EL (***H*′′**) and ER (***H*′′′**). ***I***, ***J***, Scatter plots and regression lines showing the within-trial correlation of freezing (*x*-axis) and alarm call rate (*y*-axis) for each trial (′, FC; ′′, EL; ′′′, ER), shown separately for males (***I***) and females (***J***). Correlation coefficients (*r* = Pearson’s *r*; rho = Spearman’s ρ) are shown inside each panel. *N* values: ***A–C***, ***F–J***: 20 males, 20 females; ***D***, ***E***: 20 males, 8 females (excluding animals who made no alarm calls in any trial). Dark-toned data points depict the mean ± SEM. Significant main effects of sex (+) and trial type (#) are denoted with different symbols, with 1 (*p *<* *0.05), 2 (*p *<* *0.01), or 3 (*p *<* *0.001) symbols depicting the degree of significance.

To explore how dynamically USV emissions change as the animals acquire and extinguish the CS–US association, we recorded USVs and behavior throughout FC, EL, and ER. Using a two-way repeated-measures ANOVA, we found that while the amount of baseline 50 kHz calls did not change across these trials (Fig. 6*A*,*B*), their nature changed in that some animals were observed emitting alarm calls during the baseline period (Fig. 6*C*) leading up to EL (2 of 20 males, 3 of 20 females) and ER (4 of 20 males, 0 of 20 females). Mirroring this, there was a significant main effect of trial on the latency to first alarm (mixed-effects model; effect of trial: *F*_(1.878,29.11)_ = 18.64; *p *<* *0.001; trial × sex interaction: *F*_(2,31)_ = 5.040; *p *=* *0.013; Fig. 6*D*). *Post hoc* contrasts were only significant in males (FC vs EL, *p *<* *0.001; FC vs ER, *p *<* *0.001), with a trend observed in females (FC vs EL, *p *=* *0.055). We also observed that alarm calls emitted across these trials got progressively longer (mixed-effects model; main effect of trial: *F*_(1.581,25.30)_ = 7.799; *p *=* *0.004; Fig. 6*E*) with significant *post hoc* comparisons only in males (FC vs EL, *p *=* *0.032; FC vs ER, *p *=* *0.026). The overall rate of alarm calling reduced over time (two-way repeated-measures ANOVA; main effect of trial: *F*_(1.555,59.09)_ = 12.50; *p *<* *0.001; main effect of sex: *F*_(1,38)_ = 9.534; *p *=* *0.004; Fig. 6*F*) with significant *post hoc* comparisons in males (FC vs EL, *p *=* *0.004; FC vs ER, *p *=* *0.003). Looking at the pattern of alarm calls across each trial (Fig. 6*G*′–*G*′′′), female alarm calls consistently peaked at a similar time point to males, but remained at a lower level throughout each trial [two-way repeated-measures ANOVA; main effects of sex in FC (*F*_(1,38)_ = 13.05; *p *=* *0.001), EL (*F*_(1,38)_ = 7.350; *p *=* *0.01), and ER (*F*_(1,38)_ = 4.875; *p *=* *0.033)]. During FC, we also observed higher levels of freezing in males than females (two-way repeated-measures ANOVA; main effect of sex: *F*_(1,38)_ = 6.572; *p *=* *0.014; Fig. 6*H*′). In ER, by contrast, females froze more than males (two-way repeated-measures ANOVA; main effect of sex: *F*_(1,38)_ = 7.702; *p *=* *0.009; Fig. 6*H*′′′), with no sex differences observed in EL (Fig. 6*H*′′). While male alarm call emission sloped downward during EL, it was still observed during the last 5 tones in 5 of 20 of rats (compared with only 1 female rat). The following day during ER male alarm call emission was also considerably more prevalent (12 of 20 males, 3 of 20 females), and remained so for each of the three tone presentations. Across all trials, we found significant or trending correlations between alarm call rate and freezing in males (Fig. 6*I*), but not in females (*p* values* *>* *0.658; Fig. 6*J*).

**Figure 7.**
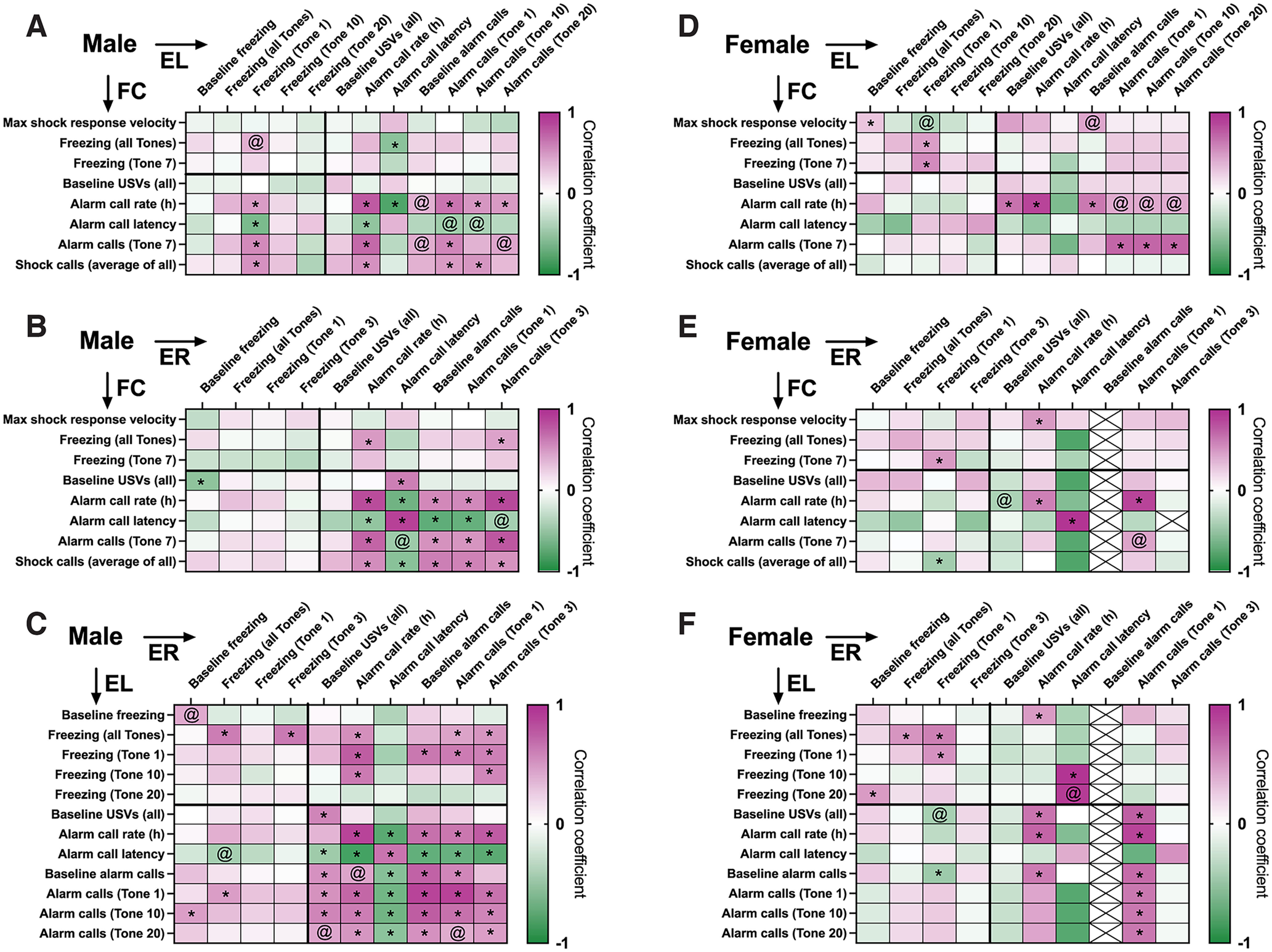
Correlation patterns of freezing and alarm call rates across fear conditioning (FC), extinction learning (EL), and extinction retrieval (ER). ***A–F***, Heatmaps showing correlation coefficients (Pearson’s or Spearman’s coefficient, depending on the distribution of the data in each variable pair) of motor and vocal behaviors during one of the trials (*y*-axis) and one of the subsequent trials (*x*-axis), separately for males (***A–C***) and females (***D***, ***E***). Color scale corresponds to correlation coefficient and direction (magenta, positive; green, negative). Significant (uncorrected, *p *<* *0.05) correlations are marked by *, trending (uncorrected, *p *<* *0.1) correlations are marked by @.

Next, we asked whether the patterns of motor and vocal behaviors in one trial correlated with motor or vocal behaviors in consequent trials. Full correlation heatmaps are presented in Figure 7. The heatmaps are split into quadrants as follows: top quadrants show motor behaviors (freezing, maximum velocity reached in response to the shock) correlated with later motor (left) or vocal behaviors (right), while bottom quadrants show vocal behaviors correlated with later motor (left) and vocal behaviors (right). In both sexes, but more notably in males, we see consistency between alarm calls across FC, EL, and ER in the form of significant correlations between alarm call rates and latencies (bottom-right quadrants; Fig. 7*A–F*). In males, we see alarm and shock call parameters in FC correlating with freezing during early EL (tone 1; Fig. 7*A*, bottom left), while in females early EL freezing is predicted by FC freezing (Fig. 7*D*, top left). Additionally, in males we observe EL freezing to be positively correlated with ER alarm call rate (Fig. 7*C*, top right).

## Discussion

Across these studies, we provide a rich picture of rat USV production during fear conditioning, demonstrating how USVs can relate to other behavioral readouts of internal states, and sex differences therein. Alarm calls in male rats were more frequent than in female rats, tracked more robustly with the intensity of the aversive experience (shock intensity), and were more resistant to extinction. Additionally, male rats that abstained from alarm vocalizations also had markedly low levels of freezing during fear conditioning. Across testing sessions, we observed significant correlations in alarm call rates in both males and females, suggesting that it may represent a trait-like individual characteristic. In males, we also saw alarm call rates and freezing correlating within and across sessions. Darting did not associate with any of the measured USV parameters in either sex. In both males and females the experience of footshocks that were not predictably preceded by a tone resulted in a delay in alarm call initiation compared with those exposed to tone-paired footshocks. We also found that in both sexes short, audible calls occurred immediately after the footshock, scaling with shock intensity. Multidimensional behavioral measurement such as this—particularly strategies that include sex and other individual differences—will push our field toward more valid and successful translational research.

We found that during the baseline period female rats emitted more short 50 kHz calls than did males, although this difference was not observed in our tone-only cohort, likely because of low power (Extended Data Fig. 2-1). These calls have been extensively reported on in appetitive circumstances (Knutson et al., 2002; Schwarting et al., 2007; Takahashi et al., 2010; Wright et al., 2010; Reinhold et al., 2019), drug exposure (Knutson et al., 1999; Wright et al., 2010; Simola, 2015; Lawson et al., 2021), and preclinical models of autism spectrum disorder (Caruso et al., 2020), and their production is modified by drug withdrawal (Lin et al., 2018), sleep deprivation, and lithium (Wendler et al., 2019). The majority of these experiments use exclusively male subjects, although some suggest that female mice are less likely to spontaneously emit such vocalizations (Michael et al., 2020). Others report that female rats emit more 50 kHz calls than males during baseline observation before fear conditioning (Willadsen et al., 2021a), when interacting with an anesthetized conspecific (Blanchard et al., 1993) and in a voluntary human handling task (Kosten et al., 2005). Our work highlights the need to include females in such experiments, at least when using a species or strain in which females reliably emit these calls, to fully understand how high-frequency calls relate to affective states and respond to interventions. It is plausible that they reflect variable states or nuances thereof across sex, and without including females the conclusions drawn will only capture part of the picture.

Shock calls spanning audible and ultrasonic ranges, akin to those recorded in mice during a tail suspension test (Ruat et al., 2022), were observed in response to the footshock in our paradigm. Such calls have been reported in female rats experiencing fear conditioning (Kosten et al., 2005; Schwarting, 2018a). Kosten et al. (2005, 2006) additionally investigated the intensity of footshock required to elicit audible vocalizations in rats and found that lower intensities were needed for females. While our large cohort did not display significant sex differences on this measure (Fig. 1*E*), in our unpaired cohort there was a main effect of sex (Fig. 3*D*) with females emitting more shock calls than males. As this finding was not replicated across our cohorts, we interpret it with caution, but where such differences have previously been observed it has been postulated to relate to sex differences in pain sensitivity (Kosten et al., 2005). Interestingly, in our dataset the number of shock calls emitted scaled with the intensity of the footshock in both males and females. Unlike alarm calls, we also observed that all rats (except for one male rat) emitted at least some shock calls. While we do not have access to the animals’ subjective perception of pain or discomfort on exposure to the shock, our findings suggest that vocalizations in direct response to painful stimuli could serve as a proxy for intensity, as opposed to alarm calls, which emerge with a delay and show sex-biased scaling with intensity. Further work should explore whether similar dose–response curves can be observed with other modalities of aversive stimuli, to determine whether this response is selective to the electric footshocks used here. Another interesting question is: are these calls modulated by pharmacological interventions targeting the pain system, such as the opioid antagonist naloxone (as shown before for alarm calls in males, Oliveira and Barros, 2006)? Our work shows the value in recording these commonly unreported vocalizations as a potential window into the experience of aversion and pain in rodents.

In this cohort, we replicated the prior finding that darting, a conditioned defensive behavior, occurs more often in females than in males, and that animals that dart have lower levels of freezing compared with Non-darters (Gruene et al., 2015; Mitchell et al., 2022). While male rodents have long dominated in preclinical samples, there has been steady improvement particularly in the behavioral field toward the inclusion of female rodents (Woitowich et al., 2020; Rechlin et al., 2022). Rigorous observation of animal behavior during standardized tests has long shown that male and female rodents may use different behavioral strategies in the face of the same threats (Blanchard et al., 1991, 1992; Blanchard, 2022). An appreciation of this concept, which has garnered revitalized attention in recent years (Shansky and Murphy, 2021), has led to exciting revelations of, for example, how accounting for sex as a biological variable can capture a broad range of ways in which the brain can handle complex problems (Chen et al., 2021). Our findings that darting occurs independently of vocal behaviors and is more common in females than males raise interesting questions. What drives these behaviors may differ between males and females, perhaps even serving variable purposes in different evolutionary niches. In natural settings, alarm calls serve to warn others of a threat (Litvin et al., 2007), but carry a risk of alerting predators to one’s location, forcing a balance between alerting others and self-preservation (or preservation of a litter of pups unable to escape). Several possibilities for sex-biased behavioral strategies and tendencies can be postulated, and future work will undoubtedly shed light on what these differences are and how we can best account for and use them for translationally valid preclinical research.

Overall, we observed that male rats made more alarm calls than females across all experiments. In males, shock intensity had a stronger effect on the total number of alarm calls emitted, as well as the latency to initiate alarm calls, than in females. Categorically, males were more likely than females to emit alarm calls, and those males that did so also froze significantly more during fear conditioning. Together, these findings point to alarm calls likely serving as a more accurate metric of negative affective state, such as perceiving a threat or experiencing discomfort, in male rats than in females. Other reports also suggest that males emit more alarm calls than females in the context of cued fear conditioning (de Vry et al., 1993; Kosten et al., 2006, 2005; Graham et al., 2009; Kassai and Gyertyán, 2012; Doncheck et al., 2020; Willadsen et al., 2021a), while early work using predator exposure found that females emitted more alarm calls than males (Blanchard et al., 1992). In studies involving only males, others found a similar dose–response relationship with shock intensity as we observe here (Wöhr et al., 2005; Hegoburu et al., 2011). Similar to our findings, these groups also reported a lack of alarm calling in some animals, along with a correlation between freezing and alarm call durations (Wöhr et al., 2005; Willadsen et al., 2021a). Here we show this correlation with freezing extends to the number of alarm calls emitted, but only in males. Our findings imply that the propensity to emit Alarm calls may be a part of a broader threat response phenotype in males, although a potential genetic basis for the Alarm caller phenotype has not yet been investigated. As the rat stock used for these experiments is outbred, the contribution of genetic variability to our findings is plausible. In an illustration of this possibility, knockout of the serotonin transporter gene reduces alarm call rates in male and female Wistar-crossed rats during fear conditioning (Willadsen et al., 2021a), and females from this strain emitted no alarm calls during extinction (Willadsen et al., 2021b). Previous work has also identified strain and sex differences in active and passive coping behaviors in repeated forced swim stress exposure (Colom-Lapetina et al., 2017), so a stock or strain effect on vocalization patterns would not be unexpected and has indeed been demonstrated (Schwarting, 2018a, b). It would also be intriguing to investigate whether Alarm callers and Non-alarm callers differ from each other in some other features, such as neuroanatomy or neuronal ensembles engaged during fear conditioning. Studying such divergent response styles could help elucidate individual differences in factors that affect response to traumatic events, and recovery thereof, with translational relevance for human psychiatric disorders.

We also observed alarm calls in several, although not all, rats throughout extinction learning and retrieval. The occurrence of these calls during an extinction or test session following fear conditioning has been reported before (Kikusui et al., 2001; Wöhr et al., 2005; Hegoburu et al., 2011; Kassai and Gyertyán, 2012) in male rats, and here we expand on this by demonstrating the pattern of alarm calls across training sessions and sexes. Others have also shown higher alarm call rates in males than females during extinction (Willadsen et al., 2021b) or a test session after fear conditioning (Kosten et al., 2006). Similar to freezing, we see alarm calls at a low level during the baseline recording time of extinction learning, with a sharp increase during tone CS presentation, followed by a gradual decay. This decay in alarm call rate was considerably steeper in females, while no differences were observed in terms of freezing during this trial. This finding highlights an important caveat in behavioral tests: our conclusions depend critically on our choice of readout. If alarm call rate was conceptualized as a measure of associative learning (discussed in more depth below), this dataset would point to a remarkable sex difference in extinction learning and retrieval, with females outperforming males on both. But if in the same animal cohort we only had access to freezing data, we would conclude that no sex differences in EL could be observed, and, if anything, the females show worse extinction retrieval than males. As it stands, our findings cannot be used to determine which behavior is more suitable for measuring internal states such as fear. Rather, we call for caution in operationalizing any internal state as a singular behavioral output. Rapid technological advances in behavioral recording and *in vivo* interrogation of neuronal activity help us take large strides toward new discoveries about what makes individuals acquire and extinguish aversive memories. However, much also remains to be done in terms of deep understanding of what the behaviors we routinely record mean, and how to best harness them for translational aims.

As certain behaviors during fear conditioning, such as darting, are known to predict later performance on extinction retrieval (Gruene et al., 2015), an important question going forward is whether alarm calling could serve as a similar predictor. In males much more strongly than in females, we observed correlations between alarm call rates and freezing both within a trial and across fear conditioning and extinction. For example, we observed that the alarm call rate during fear conditioning was positively correlated with freezing in extinction learning, but only during early trials. This could suggest that in males alarm call rate relates to the strength of association between the CS and US. However, alarm calls during fear conditioning were not related to freezing at the end of extinction learning or across extinction retrieval, suggesting this behavior may not predict the success of extinction. Our findings are somewhat in contrast to those of Willadsen et al. (2021b), who report positive correlations of the same behavior (alarm call rate, immobility time) within and across trial types, but weak or nonexisting cross-correlation of alarm call rate with immobility. The lack of association was found for both within-trial and across-trial (fear acquisition correlated with extinction training) analyses. This divergence could be because of differences in strain and analysis strategy (females and males analyzed together vs separately). We also see alarm calls in some rats during the baseline period of extinction trials, suggesting either generalization or stress sensitization, once again more so in males than in females. Additionally, in males freezing during EL correlated with the alarm call rate during ER. These findings align with the idea of behavior as a circular-causality loop, as opposed to an arc with linear and replicable outputs occurring after certain inputs (Gomez-Marin and Ghazanfar, 2019). What the animal experiences and perceives affects its behavior, and that behavior further affects what it perceives and how it behaves, all within the context of individual history and characteristics. This framework of the dynamic nature of behavior expression fits with our findings; rather than universal associations between outputs (behaviors) and inputs (experience of shocks), we observe variable relationships that are further moderated by individual factors like sex and a trait-like tendency to emit alarm calls.

An important question to consider in case of any behavior occurring in the context of aversive learning is to what extent the behavior is associative. One of the key utilities of Pavlovian conditioning is the acquisition, and later extinction, of a learned association between the US and the CS. Currently, the expression of such learning is primarily gauged by observing behavior, such as freezing and darting. However, it should be noted that no behavior specifically and exclusively denotes associative CS–US learning. Freezing, considered the gold standard of measuring associative learning, occurs in response to not just the CS but also the context in which learning has taken place (Kamprath and Wotjak, 2004), and in most standard fear-conditioning protocols it is challenging to distinguish the proportion of freezing driven by associative and nonassociative components. Freezing has also been shown in response to a CS unpaired from the US (Cossio et al., 2016; Hersman et al., 2020; Trott et al., 2022), although others report observing very little to no freezing specifically during the CS in such conditions (Maren, 2000; Maren et al., 2001). Our findings suggest that alarm calls, at least to the depth measured here, may reflect an associative learning component of fear conditioning, but by no means do so exclusively. We do see a similar pattern between alarm call emission and freezing (i.e., a gradual rise in alarm call rate as the animals experience more CS–US pairings, and a recurrence followed by gradual decay during extinction). The fact that the emission of alarm calls rarely starts after just one footshock, as previously shown by others (Reyes et al., 2021), suggests that it is not merely a response to acute discomfort, and thus could be influenced by learning in addition to continuity of the discomfort. Contextual fear conditioning also elicits alarm calls, but less so than cued fear conditioning, also supporting the notion that learning or predictability matters (Kassai and Gyertyán, 2012). Furthermore, when the association between the US and CS was reduced by carrying out unpaired fear conditioning, there was a delay in alarm call initiation, also suggesting that predictability or a learned association with a predictor may have played a role in alarm call emission. However, we also robustly observe alarm calls during unpaired fear conditioning and during ITIs regardless of CS–US pairing, indicating that they are not tied specifically to the tone, and thus not uniquely indexing the associative component of learning. Alarm call recording does not have as long a history as freezing as a measure of threat learning, and many crucial control experiments remain to be conducted such as explicit comparison between contextual and cued conditioning, sensitivity of alarm calls to habituation, and tests of long-term recall. Our data and those of others are foundational for building an understanding of how to best make use of recorded vocalization in studies of aversive memories.

While important from an ethological perspective, alarm calls may not signal the same experiences in male and female rodents. Alarm call emission in male rats is largely in line with prior literature, ergo it tracks with stimulus intensity and defensive motor behaviors. However, in female rats alarm calls were observed largely independent of stimulus intensity and defensive behaviors. Further research is needed to understand which factors within female rodents affect the nuances in USV production to best use this behavior as a readout in behavioral experiments. Significant interindividual variability (from none at all to thousands of calls within a trial) as well as intraindividual stability (as evidenced by correlation across trials) argue for studies using USVs to favor a within-subjects design, as opposed to cross-sectional approaches. Investigating the source of these individual differences, such as what makes an Alarm caller, could also be fruitful for understanding different threat or stress response types. Our findings show that USVs are a valuable, noninvasive source of data that is sensitive to experimental manipulations, but what they tell us about the affective states of animals may depend on several variables, including sex.

## Synthesis

Reviewing Editor: Erica Glasper, The Ohio State University Decisions are customarily a result of the Reviewing Editor and the peer reviewers coming together and discussing their recommendations until a consensus is reached. When revisions are invited, a fact-based synthesis statement explaining their decision and outlining what is needed to prepare a revision will be listed below. The following reviewer(s) agreed to reveal their identity: Maria Willadsen, Stefan Brudzynski.

Summary: In this manuscript, the authors examine aversive learning, where rats can exhibit many threat-related behaviors, including freezing, motor responses, and ultrasonic vocalizations (USVs). It is unclear how these behaviors related to one another. USVs were characterized in male and female rats during cued fear acquisition and extinction and sex-specific outcomes in call types were found during testing and even during extinction. These data suggest that sex differences exist in behavioral readouts of conditioned and innate threat responses. Collectively these data suggest sex-dependence in how behavioral readouts relate to innate and conditioned threat responses, and maybe even aversion. The timely paper was largely well-received by the reviewers, especially since sex differences in defensive behaviors are often a neglected in fear conditioning literature. A few concerns were raised but they should be easily addressed in a revised version of this manuscript. When appropriate, specific reviewer comment can be found in <i>italicized font</i>.

Specific Comments

Overall

• The authors are encouraged to use terminology that is commonly used within the USV literature for written text and composite figures when distinguishing between calling types, such as 50-kHz USV for “chatter calls", 22-kHz USV for “alarm calls” - and to a lesser extend audible calls for “shock calls”.

Introduction

• Lines 72-73 - The authors are encouraged to temper their language regarding the “emotional states” of men and women. Rephrasing this sentence to better represent the data which describes differences in the expression of emotions is highly suggested.

Methods

• Line 100 - dB is a relative measure. Please report from what distance the sound was recorded from the loudspeaker.

• Line 117 - Please clarify what “maximum velocity” refers to here and in other places within the manuscript (e.g., page 21, line 395).

Results

• Line 152 - Please clarify why extinction learning and retrieval were quantified using ScaredyRat and hand-scoring, while fear conditioning was only analyzed automatically. If this relates to ScaredyRat performing differently during extinction learning and retrieval as compared to fear conditioning, then please explain. Please provide the interrater-reliability between ScaredyRat and hand-scoring for extinction learning and retrieval.

• Line 185 - Please provide more detail regarding the “various types of chatter” that the rats engaged in. <i>The same comment pertains to page 11, line 207 and page 17, line 342, where it should be added in parentheses that those are 50 kHz vocalizations</i>.

• Lines 232-236 - The authors are encouraged to significantly re-write this paragraph to contain information more directly related to what is being measured in this study. For example, the paragraph starts with discussion of calls emitted from pups following maternal separation. These are not 22kHz calls and are not deemed “alarm calls”.

• Line 258 - Please note whether the call was made before or after the foot shock, as the intensity of the call may not be “typical”.

Discussion

• Key references are missing from the Discussion section.

o Lines 512-524 - Please add additional references on the sex differences in 50-kHz USV emission. <i>Willadsen et al. (2021, Prog Neuropsychopharmacol. Biol Psychiatry) found similar results of female rats emitting more 50-kHz USV in baseline periods of fear acquisition - together with confirming results for inhibited 22-kHz USV emission. Heightened 50-kHz USV emission in females was also found in social contexts (Blanchard et al. 1993, Pharmacol Biochem Behav; Kosten et al. 2005, Behav Brain Res)</i>.

o Line 526 - Please define “audible calls”. Since both shock calls and scream calls both contain audible and ultrasonic components, greater explanation is needed here.

o Lines 526-540 - Further elaboration is needed on the apparent disparity of enhanced 50-kHz USV and audible call emission with the concomitant reduction. in 22-kHz USV calling observed in females. As currently stated, the findings could be interpreted as contradictory. <i>Yet, Kosten et al. (2005, Behav Brain Res) found similar effects of sex on audible calling as response to increasing shock levels. Here, female rats showed greater senstitivity [sic] to foot shocks in terms of audible calls, with reduced 22-kHz USV emission during fear conditioning. They discussed in terms of pain sensitivity</i>. The authors should describe whether there are any indications of altered pain sensitivity in the current data?

o Line 542-564 - Please include discussion of additional reports confirming a sex-dependent effect of shock on 22-kHz USV emission, including <i>Vry et al. (1993, Eur J Pharmacol), Graham et al. (2009, Psychol Neurosci), Kosten et al. (2005, Behav Brain Res) and Kosten et al. (2006, Brain Res) reported a reduced 22-kHz USV emission in females</i>.

o Line 568 - Please add the following references related to the occurrence of 22-kHz USV during extinction: Hegoburu et al. (2011, Front Behav Neurosci) and Kassai and Gyertyán (2012, Prog Neuropsychopharmacol Biol Psychiatry). <i>Additionally, Willadsen et al. (2021, IJMS) reported (lacking) 22-kHz USV for female Wistar rats during extinction learning as compared to their 22-kHz USV emitting male conspecifics. Please add references and discuss the divergence to non-existing 22-kHz USV in Willadsen et al. (2021, IJMS) compared to your current findings</i>.

o Lines 588-607 - The authors data suggest a sex difference in darting behavior, but these findings are not discussed within the discussion. Please discuss these findings and how they relate to those found by Gruene et al. (2015, eLife). In addition, please include higher defensive behavior discussed by Blanchard et al. (1991, Neurosci Biobehav Rev). Further elaborating on these results will highlight their importance and value to the more routine measures of freezing behavior.

o Line 597 - The author state that <i>22-kHz USV may not predict success of extinction, and is in line with findings from Willadsen et al. (2021, IJMS), where 22-kHz USV during fear acquisition only correlates with freezing behavior in said phase, but not during subsequent phases of extinction and retrieval</i>. Please add a reference to support this statement.

o Line 625 - The authors state that <i>emission of 22-kHz USV rarely starts after just one foot shock, and is in line with findings from Burgdorf et al. (2000, Behav Neurosci) and Reyes et al. (2021, Neurosci Lett) where confrontation with one mild foot shock of 0.4 mA was not sufficient to induce 22-kHz USV but reduced number of 50-kHz USV</i>. Please support this statement with additional references.

Figures

• Figure 1 - The sample size is missing. To compare across all composite figures, please add the N to all, even if they remain the same as Figure 1.

• Figure 3 - In an effort to improve the readers ability to compare across figures, please indicate the shock intensity in the composite figure. Furthermore, please provide clarity on Figure 3C displaying the main effect of sex on shock calls that is not mirrored by results depicted in Figure 1E.

• Figure 5A - Please graphically indicate the results of the 2 test showing the effect of shock intensity on the frequency of 22-kHz USV.

• Figure 6 - It does not appear appropriate to compare overall calling between fear conditioning and extinction (Figure 6A) since the call types change, as described by the authors. It does, however, seem appropriate to compare 22-kHz (Figure 6B) and 50-kHz USV emission separately during fear conditioning and subsequent phases individually.

Extended Data

• Extended Data 2-1 - Please provide clarification, as the findings represented in Extended Data 2-1B are not in line with the findings from Figure 1D. Please account for this divergence within the Discussion section when referring to the sex differences in 50-kHz USV emission (512-524).

## Author Response

Synthesis Statement for Author (Required):

Summary: In this manuscript, the authors examine aversive learning, where rats can exhibit many threat-related behaviors, including freezing, motor responses, and ultrasonic vocalizations (USVs). It is unclear how these behaviors related to one another. USVs were characterized in male and female rats during cued fear acquisition and extinction and sex-specific outcomes in call types were found during testing and even during extinction. These data suggest that sex differences exist in behavioral readouts of conditioned and innate threat responses. Collectively these data suggest sex-dependence in how behavioral readouts relate to innate and conditioned threat responses, and maybe even aversion. The timely paper was largely well-received by the reviewers, especially since sex differences in defensive behaviors are often a neglected in fear conditioning literature. A few concerns were raised but they should be easily addressed in a revised version of this manuscript. When appropriate, specific reviewer comment can be found in italicized font.

We thank all reviewers and the editor for the thorough evaluation of our manuscript. We are especially grateful for the suggested additional references which were omitted from the previous version; they have added significantly to our discussion about how this work relates to existing literature. Please find comments addressing the specific concerns below, and notes on how the manuscript was edited to take these suggestions into account. Note that the line numbers in the edited version differ from those in the original submission due to altered format (figures provided as separate files rather than embedded into the text file as requested by the editorial team).

Specific Comments

Overall

• The authors are encouraged to use terminology that is commonly used within the USV literature for written text and composite figures when distinguishing between calling types, such as 50-kHz USV for “chatter calls", 22-kHz USV for “alarm calls” - and to a lesser extend audible calls for “shock calls”.

We appreciate this call to harmonize the terminology with existing literature. We have added a clarification in the abstract regarding the alarm calls being 22 kHz, and in the Introduction,

Methods and Results we explain what the term “alarm call” refers to within this article. We have found in discussing this data with colleagues that the term “alarm call” is clearer and more accessible than “22 kHz call", which has led to our preference to use it throughout. As for 50 kHz calls, we have substituted this term instead of the previously-used “chatter call” in text, figure legends and figure panels. Finally, as the shock calls also have a distinct ultrasonic component, we feel that “audible call” does not sufficiently describe them, but have added clarifications in text (e.g. line 190).

Introduction

• Lines 72-73 - The authors are encouraged to temper their language regarding the “emotional states” of men and women. Rephrasing this sentence to better represent the data which describes differences in the expression of emotions is highly suggested.

This part of the introduction discusses preclinical rodent work, which has largely built solely on experiments using male rodents, and was not intended to imply generalizability directly to human men and women. We have added some clarifications to this sentence.

Methods

• Line 100 - dB is a relative measure. Please report from what distance the sound was recorded from the loudspeaker.

Decibel readings were recorded using a small portable recorder placed in the center of the conditioning chamber, approximately 20 cm from the speaker. We have added a note of this in the Methods section (lines 99-100).

• Line 117 - Please clarify what “maximum velocity” refers to here and in other places within the manuscript (e.g., page 21, line 395).

Maximum velocity refers to the fastest speed (cm/s) the animal is recorded to reach during the 5 second period immediately following and including shock delivery as in previous work by Gruene et al 2015 and Mitchell et al 2022. This has been clarified in the positions pointed out as well as relevant figure legends.

Results

• Line 152 - Please clarify why extinction learning and retrieval were quantified using

ScaredyRat and hand-scoring, while fear conditioning was only analyzed automatically. If this relates to ScaredyRat performing differently during extinction learning and retrieval as compared to fear conditioning, then please explain. Please provide the interrater-reliability between ScaredyRat and hand-scoring for extinction learning and retrieval.

It is well known among users of automated motion-based scoring software that when animals are sleeping, the software typically assigns this as freezing. During long extinction learning protocols such as ours we observe that rats freeze during early tone presentations, but very often fall asleep during later tones. Thus, while ScaredyRat performs very well compared to experimenter hand scoring for fear conditioning trials (as validated in prior publications), for extinction learning trials we would get erroneously high freezing data for the time the animals spend asleep if relying solely on ScaredyRat. These two states can be readily distinguished by trained experimenters (by observing for example posture and reactions to tone onset/end), which is why the latter halves of extinction learning trials are hand-scored. An explanation of this has been added to Methods.

• Line 185 - Please provide more detail regarding the “various types of chatter” that the rats engaged in. The same comment pertains to page 11, line 207 and page 17, line 342, where it should be added in parentheses that those are 50 kHz vocalizations.

We have provided the suggested clarification in all positions of the manuscript that previously referred to “chatter calls", as well as in Figure 1 D.

• Lines 232-236 - The authors are encouraged to significantly re-write this paragraph to contain information more directly related to what is being measured in this study. For example, the paragraph starts with discussion of calls emitted from pups following maternal separation. These are not 22kHz calls and are not deemed “alarm calls”.

This section has been rewritten to focus on adult alarm calls (references to pup calls omitted).

• Line 258 - Please note whether the call was made before or after the foot shock, as the intensity of the call may not be “typical”.

The representative example alarm call in this figure was captured from an intertrial interval period, and is shown here for illustrative purposes only. We have added a note on this in the figure legend. No such calls were in any animals observed before the delivery of shocks, or in animals receiving no shocks (see tone-only animals in Extended Data 2-1 C). We have refrained from analyzing call intensity for these experiments due to technical limitations, but this is a typical alarm call among the thousands we observed in total, both in terms of length and approximate intensity.

Discussion

• Key references are missing from the Discussion section.

o Lines 512-524 - Please add additional references on the sex differences in 50-kHz USV emission. Willadsen et al. (2021, Prog Neuropsychopharmacol. Biol Psychiatry) found similar results of female rats emitting more 50-kHz USV in baseline periods of fear acquisition - together with confirming results for inhibited 22-kHz USV emission. Heightened 50-kHz USV emission in females was also found in social contexts (Blanchard et al. 1993, Pharmacol Biochem Behav; Kosten et al. 2005, Behav Brain Res).

The suggested references have been added into the relevant parts of the Discussion.

o Line 526 - Please define “audible calls”. Since both shock calls and scream calls both contain audible and ultrasonic components, greater explanation is needed here.

We have added a clarification for this section. Of note, alarm calls (which we presume is what is referred to by “scream calls”) generally do not have audible components to them as the lowest frequency occurs at around 22 kHz.

o Lines 526-540 - Further elaboration is needed on the apparent disparity of enhanced 50- kHz USV and audible call emission with the concomitant reduction. in 22-kHz USV calling observed in females. As currently stated, the findings could be interpreted as contradictory. Yet, Kosten et al. (2005, Behav Brain Res) found similar effects of sex on audible calling as response to increasing shock levels. Here, female rats showed greater senstitivity [sic] to foot shocks in terms of audible calls, with reduced 22-kHz USV emission during fear conditioning. They discussed in terms of pain sensitivity. The authors should describe whether there are any indications of altered pain sensitivity in the current data?

We thank the reviewers for pointing out this finding in the literature, and have added a discussion of it. In this section of the Discussion (lines 392 -398) we discuss audible and shock calls in the context of pain experience, which admittedly is a difficult matter due to its subjectivity. As noted in the response to comments regarding Figure 3 (panel C in particular), we do not feel that our data provides strong support for sex differences in pain sensitivity as indexed by the number of emitted shock calls, and have thus exercised caution in discussing it.

o Line 542-564 - Please include discussion of additional reports confirming a sex-dependent effect of shock on 22-kHz USV emission, including Vry et al. (1993, Eur J Pharmacol), Graham et al. (2009, Psychol Neurosci), Kosten et al. (2005, Behav Brain Res) and Kosten et al. (2006, Brain Res) reported a reduced 22-kHz USV emission in females.

We have added these references to the Discussion.

o Line 568 - Please add the following references related to the occurrence of 22-kHz USV during extinction: Hegoburu et al. (2011, Front Behav Neurosci) and Kassai and Gyertyán (2012, Prog Neuropsychopharmacol Biol Psychiatry). Additionally, Willadsen et al. (2021, IJMS) reported (lacking) 22-kHz USV for female Wistar rats during extinction learning as compared to their 22-kHz USV emitting male conspecifics. Please add references and discuss the divergence to non-existing 22-kHz USV in Willadsen et al. (2021, IJMS) compared to your current findings.

We have added these references to support our discussion about sex differences in extinction. As noted in papers using multiple rat strains, the genetic background of the rats can impact vocalization behavior as well, and we think this could be a reason for the differences observed in our dataset (Sprague-Dawley rats) and the work from Willadsen and colleagues (Wistar-crossed genetic mutants). We have added a mention of this in an earlier part of the Discussion where we discuss the potential influence of genetic background (line 454-458).

o Lines 588-607 - The authors data suggest a sex difference in darting behavior, but these findings are not discussed within the discussion. Please discuss these findings and how they relate to those found by Gruene et al. (2015, eLife). In addition, please include higher defensive behavior discussed by Blanchard et al. (1991, Neurosci Biobehav Rev). Further elaborating on these results will highlight their importance and value to the more routine measures of freezing behavior.

We thank the reviewers for pointing out the need to expand on the discussion of sex differences in behavioral repertoires. To give readers more context of how sex differences in darting and its relation to vocal behaviors fit in with prior work, we have added a new paragraph covering historical and recent works (lines 413-432).

o Line 597 - The author state that 22-kHz USV may not predict success of extinction, and is in line with findings from Willadsen et al. (2021, IJMS), where 22-kHz USV during fear acquisition only correlates with freezing behavior in said phase, but not during subsequent phases of extinction and retrieval. Please add a reference to support this statement.

We actually find, as shown in Figure 7, that alarm call rate and latency during FC do correlate with freezing in a later EL session, but only in males. Interestingly, this correlation was only seen when looking at freezing during the early parts of EL (see top-left quadrant of top-left correlation matrix). This, to us, suggests that in males, alarm call rate during FC can serve as a index of memory of the CS-US association as retrieved at the start of EL (as discussed on lines 495-496). This association was not seen in females, where we saw within-behavior correlation in both freezing and alarm call rat across trials, but no strong correlations across behavioral modalities.

Our findings thus do not align with those in the aforementioned paper. We postulate that this could be due to several factors, such as males and females being analyzed together and the rat strain being different. We have added some discussion of these points on lines 498-504.

o Line 625 - The authors state that emission of 22-kHz USV rarely starts after just one foot shock, and is in line with findings from Burgdorf et al. (2000, Behav Neurosci) and Reyes et al. (2021, Neurosci Lett) where confrontation with one mild foot shock of 0.4 mA was not sufficient to induce 22-kHz USV but reduced number of 50-kHz USV. Please support this statement with additional references.

References to these works have been added to the manuscript.

Figures

• Figure 1 - The sample size is missing. To compare across all composite figures, please add the

N to all, even if they remain the same as Figure 1.

Group sample sizes have been added to the Figure Legend.

• Figure 3 - In an effort to improve the readers ability to compare across figures, please indicate the shock intensity in the composite figure. Furthermore, please provide clarity on

Figure 3C displaying the main effect of sex on shock calls that is not mirrored by results depicted in Figure 1E.

All animals included in Figure 3 received the moderate foot shock intensity (0.5 mA), which we have clarified in the figure and legend. As pointed out, in this cohort we did see a small effect of sex on shock calls (females emitting more shock calls than males, within-condition post hoc comparisons were not significant), one that was not seen in the larger cohort used for Figure 1. This divergence can be due to multiple factors, such as variance between cohorts (rats used for Figure 1 and Figure 3 are fully non-overlapping as stated in Methods) and method of analysis (Figure 1 E analysis accounts for shock intensity). As the main effect of sex for Figure 1E did not approach significance (p = 0.779) we did not think it appropriate to conduct within-shock-intensity posthoc tests to compare the sexes. To give an idea of how these data sets compared, we conducted a simple t-test comparing sexes within just the 0.5 mA group from Figure 1E, where females do again emit slightly more shock calls than males but not significantly so (p = 0.121). Due to the lack of replicating this result across our own experiments, we exercise caution in its interpretation and chose not to make statements about a potential sex difference in shock calling within specific shock intensity conditions.

• Figure 5A - Please graphically indicate the results of the 2 test showing the effect of shock intensity on the frequency of 22-kHz USV.

Results of the chi-squared tests are now indicated in both Figures 4 and 5 (effect of shock intensity on categorization of animals into Darters vs Non-darters, and Alarm callers vs Non alarm callers, respectively).

• Figure 6 - It does not appear appropriate to compare overall calling between fear conditioning and extinction (Figure 6A) since the call types change, as described by the authors. It does, however, seem appropriate to compare 22-kHz (Figure 6B) and 50-kHz USV emission separately during fear conditioning and subsequent phases individually.

We have added a new panel B into Figure 6 showing the number of 50 kHz calls emitted during the baseline periods of each three paradigms. The data shows a general downward slope, but neither the main effect of Sex or Trial nor their interaction term reached significance.

Extended Data

• Extended Data 2-1 - Please provide clarification, as the findings represented in Extended

Data 2-1B are not in line with the findings from Figure 1D. Please account for this divergence within the Discussion section when referring to the sex differences in 50-kHz USV emission (512-524).

Similarly to the question regarding non-replication of a difference between Fig 3C and Fig 1E, the data for Fig 1D and Extended Data 2-1B come from two non-overlapping cohorts of rats.

The experiment conducted for Extended Data 1-2B was not powered to detect a sex difference on any measure but rather the occurrence of different types of calls. Thus the lack of observing the effect from Fig 1D in Extended Data 1-2B is most likely due to a power issue.

We have added a note of this in the Discussion (line 372).
