# Supplementary figures and images for: Sounding the Alarm: Sex Differences in Rat Ultrasonic Vocalizations during Pavlovian Fear Conditioning and Extinction
Source: eNeuro. 2022 Dec 15;9(6):ENEURO.0382-22.2022. doi: 10.1523/ENEURO.0382-22.2022 (PMC9797209; doi:10.1523/ENEURO.0382-22.2022)

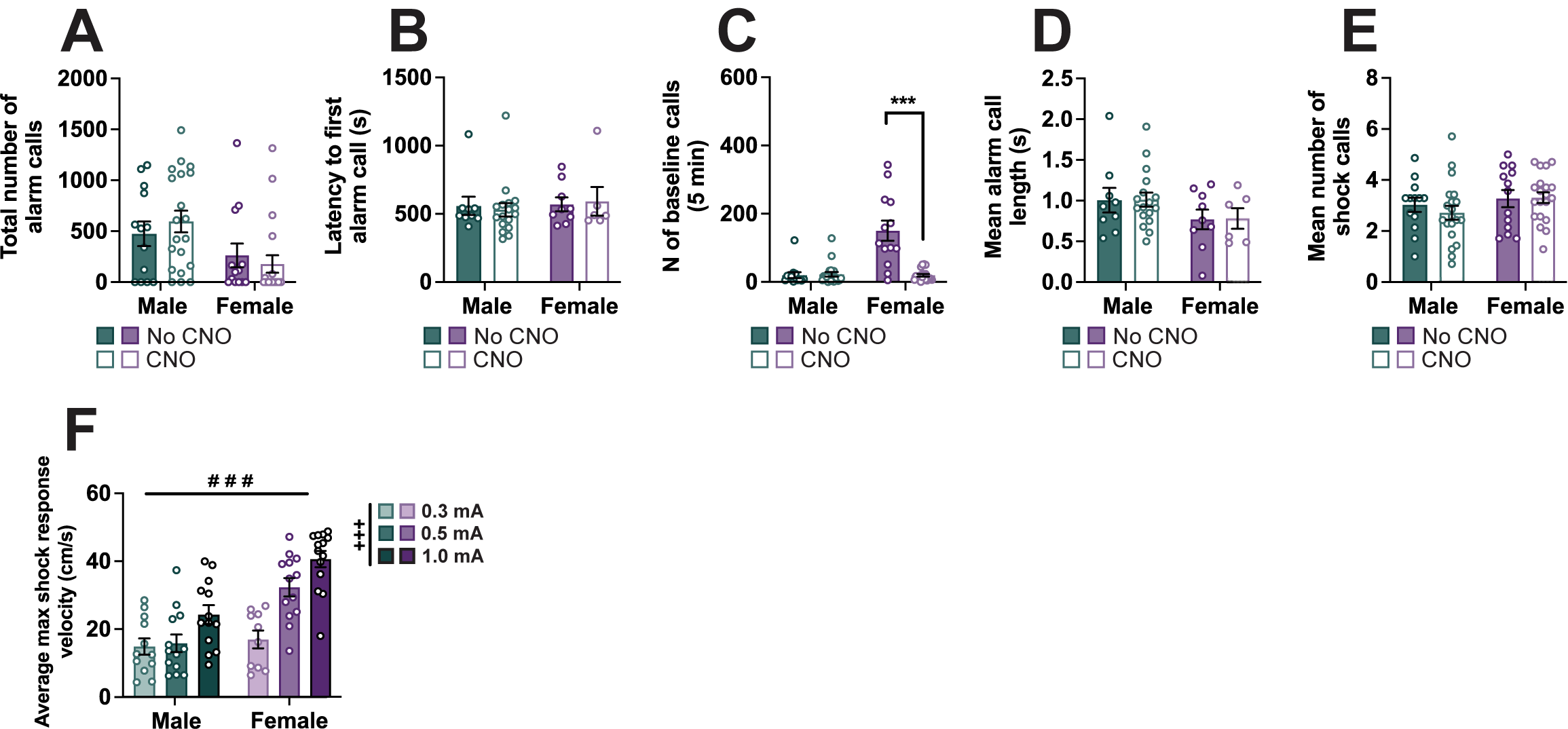

Supplement: Figure 1-1 — CNO exposure does not affect alarm call parameters in males or females. A–E, Bar graphs depicting the total number of alarm calls emitted during fear conditioning (A), the latency to the first alarm call during the session (B), the number of all calls emitted during the baseline period (C), the mean alarm call length (D), and the average number of shock calls emitted per animal across all 7 shocks (E) of male and female rats exposed to 0.5 mA footshocks, compared between experiments (one involving CNO injection and one with no injections). F, Bar graphs depicting the average maximum velocity reached immediately after shock delivery, split by sex and shock intensity. Bar graphs depict the mean ± SEM, and each dot represents a single animal. Significant main effects of shock intensity (+) and sex (#), and post hoc comparisons (*) are denoted with different symbols, with 1 (p < 0.05), 2 (p < 0.01), or 3 (p < 0.001) symbols depicting the degree of significance. Download Figure 1-1, file. [file enu-eN-NWR-0382-22-s02.tif]

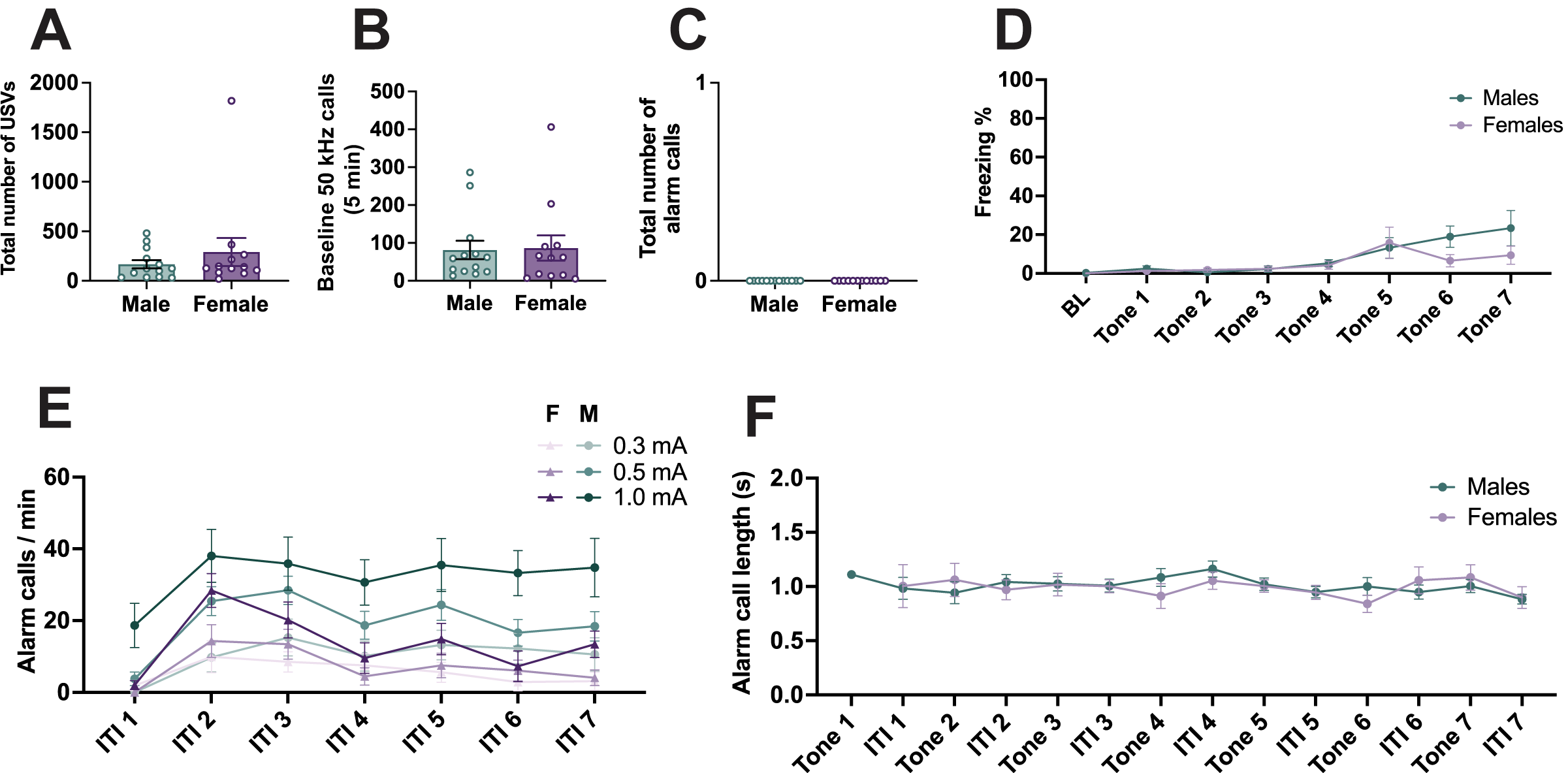

Supplement: Figure 2-1 — Mere exposure to the experimental context and tone are not sufficient to produce alarm calls, and shock-driven alarm calls are also observed during the ITIs. A–C, Bar graph showing the total number of USVs across the whole trial (A), the total number of USVs emitted during the baseline (B), and the total number of alarm calls (C) emitted by male and female rats exposed to the same testing procedure, chamber, and tones as those in Figure 1, but with no footshocks. D, Line graph showing the percentage of time spent freezing during baseline (first 2 min only) and each tone of animals exposed to only the tones without shocks. E, Line graph depicting the rate of alarm calling of male and female rats in each shock intensity group (0.3, 0.5, and 1 mA) during the ITIs. F, Line graph showing the mean alarm call length as measured during each tone and ITI, separately for males and females. N values: A–D: 13 males, 12 females; E: 67 males (0.3 mA, 21; 0.5 mA, 33; 1 mA, 13), 67 females (0.3 mA, 20; 0.5 mA, 33; 1 mA, 14); F: 50 males, 37 females (non-alarm callers excluded). Bar graphs depict the mean ± SEM, and each dot represents a single animal. Symbols along line graphs indicate the mean ± SEM. Download Figure 2-1, file. [file enu-eN-NWR-0382-22-s03.tif]

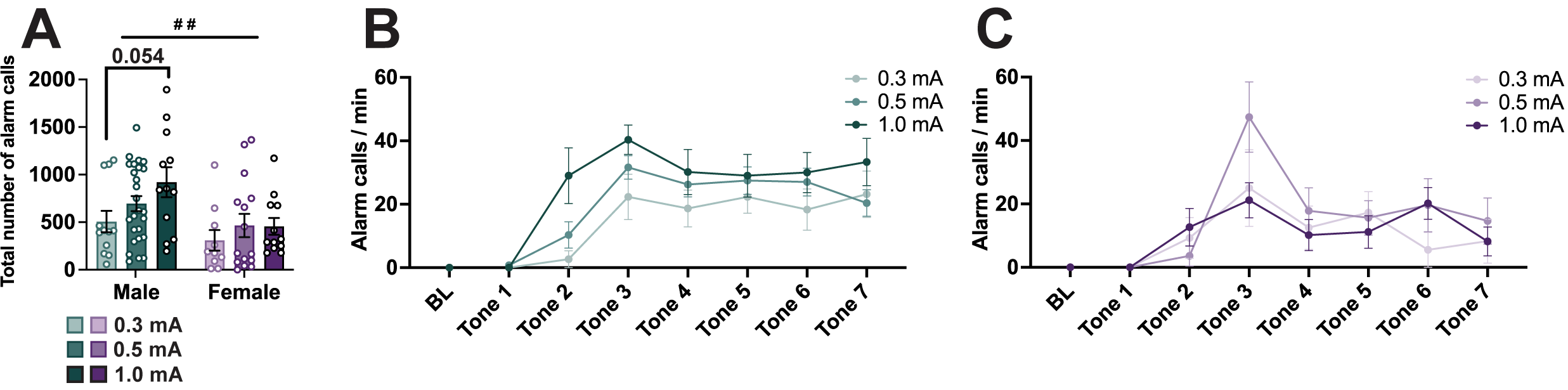

Supplement: Figure 5-1 — Removing non-alarm callers does not eliminate the sex difference in alarm call rate. A, Total number of alarm calls emitted by rats included in Figure 2C, excluding rats that did not make any alarm calls. B, C, Line graphs showing the normalized (per minute) alarm call rate of male (B) and female (C) rats across shock intensity groups during baseline (BL; 5 min) and each tone, excluding rats that did not make any alarm calls. N values: 50 males (0.3 mA, 12; 0.5 mA, 26; 1 mA, 12), 37 females (0.3 mA, 10; 0.5 mA, 15; 1 mA, 12). Bar graphs depict the mean ± SEM, and each dot represents a single animal. Symbols along line graphs indicate the mean ± SEM. Significant main effects of shock intensity (+) and sex (#), and post hoc comparisons (*) are denoted with different symbols, with 1 (p < 0.05), 2 (p < 0.01), or 3 (p < 0.001) symbols depicting the degree of significance. Download Figure 5-1, TIF file. [file enu-eN-NWR-0382-22-s04.tif]
